# EZH2‐Mediated Epigenetic Modifications Induce Pyroptosis in Dental Pulp Endothelial Cells via Activation of NLRP6 Inflammasome During Pulpitis Development

**DOI:** 10.1155/mi/5876995

**Published:** 2026-04-08

**Authors:** Weilin Zhou, Weili Huang, Hongjing You, Zhixi Huang, Jingye Zhou, Yi Huang

**Affiliations:** ^1^ Hospital of Stomatology, The First Affiliated Hospital, Clinical Research Platform for Interdiscipline, School of Stomatology, Jinan University, Guangzhou, China, jnu.edu.cn; ^2^ School of Pharmaceutical, Guangzhou University of Chinese Medicine, Guangzhou, China, gzucm.edu.cn

**Keywords:** EZH2, inflammasome, NLRP6, pulpitis, pyroptosis, vascular endothelial cell

## Abstract

**Objectives:**

Recent studies suggest that alleviating pyroptosis may be an effective strategy for treating inflammation. This research explores the changes in cellular composition and pyroptotic pathways in dental pulp tissue during pulpitis and preliminarily validates the regulatory role of EZH2 in lipopolysaccharide (LPS)‐induced pyroptosis of vascular endothelial cells (ECs) via NLRP6, aiming to provide a theoretical foundation for understanding pulpitis mechanisms.

**Methods:**

Bioinformatics analysis of public single‐cell RNA sequencing (scRNA‐seq) and microarray data, together with RNA‐seq of EZH2^fl/fl^;Tie2‐Cre^+/−^ pulp tissue, was performed to investigate the involvement of ECs and pyroptosis in pulpitis. Functional validation was conducted using an EZH2‐inhibited inflammatory model and an NLRP6 knockdown model.

**Results:**

Bioinformatics analysis revealed a strong association between pulpitis and EC pyroptosis. RNA‐seq further demonstrated that EZH2 deficiency was associated with enhanced pyroptotic responses in ECs. In vitro, EZH2 inhibition or NLRP6 knockdown markedly attenuated LPS‐induced inflammasome activation and pyroptosis in EOMA cells. This illustrates that the EZH2/NLRP6 axis plays a critical regulatory role in EC pyroptosis and suggests its potential as a therapeutic target in pulpitis.

**Conclusions:**

In summary, this study establishes ECs as key regulators in pulpitis inflammation, highlighting EZH2’s role in modulating NLRP6 and the potential mechanism of pyroptosis in ECs.

## 1. Introduction

The vascular richness of dental pulp is comparable to that of the brain, which is among the most vascularized areas in the body [1]. Notably, its average capillary density is higher than in most other tissues, and blood flow is elevated. These features endow the pulp with a strong capacity for injury resistance and self‐repair [2–5]. During pulpitis, a localized inflammatory disease of the oral cavity, the pulp vasculature undergoes dynamic structural and functional changes [6]. These alterations include vasodilation, increased vascular permeability, and elevated intrapulpal pressure. Together, they enhance circulation, facilitate the clearance of harmful substances, and accelerate the resolution of inflammation [7]. These vascular changes are primarily mediated by endothelial cells (ECs), which, as the principal structural components of the vascular wall, regulate intrapulpal pressure, prevent leakage of blood constituents, and modulate local immune responses [8–10]. In response to ongoing inflammation that becomes chronic, ECs may promote capillary remodeling and neovascularization by releasing vascular endothelial growth factor (VEGF), thereby contributing to pulp tissue repair [11, 12]. Among the potential molecular mechanisms underlying EC regulation of pulpitis, epigenetic modifications have been implicated, which may reveal targets for early intervention to prevent disease progression and restore pulp vitality.

Epigenetic modifications, such as CpG DNA methylation, histone post‐translational modifications, and noncoding RNA expression, have been implicated in inflammasome regulation [13]. Dysregulation of these processes can contribute to inflammatory disorders, neurological diseases, and cancer [14]. Among these regulators, enhancer of zeste homolog 2 (EZH2), a histone methyltransferase, catalyzes trimethylation of histone H3 at lysine 27 (H3K27me3), leading to chromatin compaction and transcriptional repression [15]. EZH2 is highly expressed in ECs, and emerging evidence suggests that it may modulate the progression of pulpitis by regulating inflammasome activity, thereby influencing oral disease development [16, 17]. Specifically, in infected dental pulp, EZH2 suppresses the proliferation of human dental pulp cells (HDPCs) by reducing the expression of inflammatory genes through H3K27me3 modification, ultimately affecting cell cycle progression and apoptosis [18]. Notably, EZH2 can be recruited to the promoter region of pyrin domain‐containing nucleotide‐binding oligomerization domain‐like receptor protein 6 (NLRP6), resulting in increased H3K27me3 deposition and suppression of NLRP6 transcription [19].

NLRP6, as a cytosolic supramolecular complex, plays a critical role in recognizing pathogen invasion and regulating immune responses [20, 21]. These functions are mediated through assembly of the NLRP6 inflammasome, which activates downstream caspases and cleaves gasdermin D (GSDMD), ultimately triggering pyroptosis, a form of cell death characterized by cellular swelling, membrane rupture, cytoplasmic release, and inflammatory cytokine secretion [22–25]. The NLRP6 inflammasome can be categorized into typical and atypical forms. In the typical pathway, activated NLRP6 recruits apoptosis‐related speck‐like protein (ASC, *PYCARD*) and Pro‐Caspase‐1 to assemble a platform that enables Caspase‐1 activation [26]. In the atypical pathway, NLRP6 instead activates Caspase‐11 in mice and Caspase‐4/5 in humans [27, 28]. These caspases further process pro‐inflammatory cytokines such as Interleukin‐1β (IL‐1β) and pro‐IL‐18 into their mature forms [27]. Through these mechanisms, NLRP6 thereby contributes to pathogen clearance and the maintenance of immune homeostasis. Importantly, NLRP6 is expressed in vascular ECs of healthy dental pulp and tends to be upregulated in inflamed pulp tissues, suggesting that ECs may serve as a key site for inflammasome activity [29, 30]. However, whether EZH2 modulates NLRP6 inflammasome activity in ECs during pulpitis remains to be investigated.

In summary, vascular ECs in the dental pulp may play a pivotal role in inflammation and pyroptosis. The NLRP6 inflammasome is a key mediator of this process, and EZH2 is likely to influence its activity in pulp ECs. However, the precise mechanisms remain unclear. Elucidating this potential regulatory network will not only enhance our understanding of pulpitis pathogenesis but also provide novel insights for early therapeutic intervention.

## 2. Materials and Methods

### 2.1. Data Collection

All datasets analyzed in this study were obtained from the Gene Expression Omnibus (GEO) database (https://www.ncbi.nlm.nih.gov/geo). In total, four transcriptomic datasets related to human dental pulp were included, comprising three single‐cell RNA sequencing (scRNA‐seq) datasets with seven samples of healthy dental pulp and one microarray‐based gene expression dataset with six healthy and six irreversible pulpitis samples, with clinical diagnosis based on the criteria of the American Association of Endodontists (AAE). Specifically, the scRNA‐seq datasets were GSE164157 (GSM4998457–GSM4998461), GSE185222 (GSM5608427), and GSE146123 (GSM4365602). The microarray dataset was GSE77459 (GSM2052371–GSM2052382). Commonly used cell type marker gene lists were collected from published literature and various cell type databases, including CellMarker, CellAtlas, and the Human Cell Atlas, to provide foundational data for annotating dental pulp cells (Tables S1 and S2). Additionally, histone methylation‐related genes were collected from the EpiFactors database (https://epifactors.autosome.org) and filtered by functional roles and involvement in key histone‐modifying complexes, with the 19 most relevant genes selected for downstream analysis (Table S3). A total of 33 pyroptosis‐related genes (PRGs) were obtained from the literature, and corresponding pyroptosis pathways were collected from the KEGG database (Table S4) [31].

### 2.2. Preprocessing of Public scRNA‐seq Data

All scRNA‐seq data were processed in the Seurat R package with default parameters unless otherwise specified. Raw sequencing data were quality‐checked with FastQC, and cells with a Phred quality score of less than 20 or fewer than 5000 detected transcripts were excluded; mitochondrial genes were removed prior to downstream analyses. Filtered data were normalized using the LogNormalize method (scale factor = 10,000), and 2000 highly variable genes were selected using the Variance Stabilizing Transformation (VST) method. Principal component analysis (PCA) was performed on these features, and the top 10 principal components were used for nearest‐neighbor graph construction, followed by Louvain clustering at a resolution of 0.8. Doublets were identified using DoubletFinder (pN = 0.25, pK automatically determined, nExp estimated at ~5% of total cells) and validated through UMAP visualization. To minimize nonbiological batch effects across datasets, we applied the Harmony algorithm (group.by.vars = "dataset") after data normalization and dimensionality reduction. This approach generated a unified low‐dimensional embedding, thereby reducing dataset‐specific biases and enabling reliable cross‐dataset comparisons.

### 2.3. Cell Type and Subtype Annotation of Public scRNA‐seq Data

The R tool Cell Type Identification by Estimating Relative Subsets of RNA Transcripts expressed by high‐throughput sequencing (CHETAH) (https://www.humancellatlas.org) was employed to predict the cell type for each cell and perform preliminary annotations based on the marker gene lists. To visualize the spatial distribution of different cell types and subtypes, dimensionality reduction was conducted using the UMAP method, which preserves cell similarity in high‐dimensional space for clearer representation in the low‐dimensional embedding plot. After completing the preliminary annotation and dimensionality reduction analysis, cross‐validation of the CHETAH annotation results with the collected marker gene data assessed annotation accuracy. For significantly differing annotation results, further literature reviews and manual adjustments were performed to enhance accuracy and reliability. Finally, visualization tools were utilized to present the analysis results, clearly displaying the distribution of different cell types and EC subpopulations. In addition, the FeaturePlot function was applied in Seurat to visualize the expression of PRGs within EC subpopulations on the UMAP embedding.

### 2.4. Differential and Correlation Analyses

Differential gene expression analysis for GSE77459 was performed at the probe level using the GEO2R online tool. Probe annotations were obtained from the platform file to map probe IDs to gene symbols; multiple probes mapping to the same gene were averaged, and probes mapping to multiple genes were excluded. Differentially expressed genes (DEGs) were identified with adjusted *p*‐value (*P*_adj_) < 0.05 and log2|fold change (FC)| ≥ 2. Gene set enrichment analysis (GSEA) was conducted using the R package clusterProfiler on the processed expression data. Pyroptosis‐related pathways yielded a defined gene set (S), and the GSE77459 expression data served as the expression matrix (L), with a normalized enrichment score (NES) > 1 and *P*_adj_ < 0.05 indicating significant enrichment. EC marker genes combined with PRGs were analyzed in the STRING database (https://cn.string-db.org) to construct a protein–protein interaction (PPI) network (interaction score ≥ 0.400), which was subsequently visualized and optimized using Cytoscape. Pearson correlation analysis was performed to assess associations among histone methylation‐related genes, DEGs, and PRGs, with additional evaluation of correlations between PRGs and EZH2. Visualization of correlation patterns was performed using the corrplot R package, and genes with an absolute correlation coefficient |*r*| > 0.5 and *p*  < 0.05 were considered statistically significant.

### 2.5. Tissue Preparation

EC‐specific EZH2 conditional knockout mice (EZH2^fl/fl^;Tie2‐Cre^+/−^ mice) were generated by crossing EZH2^fl/fl^ mice with Tie2‐Cre transgenic mice and were kindly provided by Professor Yang Chen (School of Pharmaceutical, Guangzhou University of Chinese Medicine, Guangzhou, China). All procedures involving mammalian subjects were conducted in strict accordance with ethical guidelines for animal research and approved by the Ethics Committee for Experimental Animals at the School of Pharmaceutical Sciences, Guangzhou University of Chinese Medicine (Animal Facility License Number: SYXK (Yue) 2019‐0202). Mouse genotyping and dental pulp tissue collection were performed according to the procedures described in our previous study [32]. Dental pulp tissues from three EZH2^fl/fl^;Tie2‐Cre^+/−^ mice (male, 3 months old, 25 ± 5g; conditional knockout group, CKO) and three wild‐type mice (WT) were harvested. All samples were immediately snap‐frozen in liquid nitrogen and stored at −80°C until RNA sequencing.

### 2.6. RNA‐seq

RNA integrity (RIN value) and purity (OD260/280 and OD260/230 ratios) were assessed using the Agilent 2100 Bioanalyzer. Total RNA was subjected to mRNA enrichment, followed by double‐stranded cDNA synthesis, purification, fragment size selection using AMPure XP beads, and PCR amplification to construct sequencing libraries. Library quality was assessed prior to sequencing on the Illumina NovaSeq 6000 platform. Raw sequencing reads were assessed for quality using FastQC. Low‐quality reads and sequencing artifacts were filtered to obtain clean reads, which were subsequently aligned to the mouse reference genome (GRCm39, Ensembl release 106) using HISAT2. Gene expression levels were quantified, and DEGs were identified using the criteria of log2|FC| ≥ 2 and *P*_adj_ < 0.05. GO functional analysis and KEGG pathway enrichment of DEGs were performed using DAVID. Differentially expressed PRGs were visualized using a heatmap generated with the pheatmap package in R.

### 2.7. Cell Culture and Treatments

Endometrial mesenchymal stromal (EOMA) cells were obtained from Guangzhou Jennio Biotech Co., Ltd. (ATCC, USA). EOMA cells were cultured in Dulbecco’s modified Eagle medium (DMEM) supplemented with 10% heat‐inactivated fetal bovine serum, 100 U/mL penicillin, and 100 µg/mL streptomycin, in a humidified incubator at 37°C with 5% CO_2_. Cells were stimulated with *Escherichia coli* lipopolysaccharide (LPS; Sigma, USA, Cat. Number L4130) at 0.5, 1, or 2 µg/mL for 24 h to establish an inflammatory environment, with 5 µM ATP (Sigma, USA, Cat. Number A2383) during the last 4 h. For inhibitor assays, cells were treated with GSK126 (MCE, China, Cat. Number HY‐13470) for 48 h together with 2 µg/mL LPS and ATP.

### 2.8. Viral Transduction

Lentiviral vectors encoding *Nlrp6*‐targeting shRNA were constructed, with a scrambled shRNA vector used as a negative control. EOMA cells were plated in six‐well plates at 8 × 10^4^ cells per well and infected with the lentivirus at a multiplicity of infection of 15 for 72 h according to the manufacturer’s instructions. Cells stably expressing the shRNA were subsequently selected using 5 μg/mL puromycin.

### 2.9. Western Blot Analysis

Total protein was extracted using RIPA buffer (Thermo, USA) and quantified using the BCA Protein Assay Kit (Beyotime, China). Equal amounts of protein were separated by 10% SDS‐PAGE and transferred onto 0.2 μm polyvinylidene fluoride (PVDF) membranes (Millipore, USA). Membranes were blocked and incubated overnight at 4°C with primary antibodies. The primary antibodies were anti‐EZH2 (1:1500; CST, USA, Cat. Number 5246s), anti‐NLRP6 (1:1500; Novus, USA, Cat. Number NBP2‐31372), anti‐Caspase‐1 (1:1000; Santa, USA, Cat. Number sc‐56036), anti‐ASC (1:2000; Santa, USA, Cat. Number sc‐514414), anti‐Pro‐IL‐1*β* (1:500; Wanlei, China, Cat. Number WLH3903), anti‐IL‐1*β* (1:500; Wanlei, China, Cat. Number WL00891), anti‐IL‐18 (1:500; Wanlei, China, Cat. Number WL01127), anti‐GSDMD (1:1000; Abcam, UK, Cat. Number ab209845), and anti‐β‐actin (1:1000; CST, USA, Cat. Number 4967) was used as an internal control. The secondary antibodies were anti‐mouse IgG (1:1500; CST, USA, Cat. Number 7076s) and anti‐rabbit IgG (1:1500; CST, USA, Cat. Number 7074s). The target bands were detected by an automatic chemiluminescence/fluorescence ImageJ analyzer (Tanon, China) and analyzed using ImageJ software. All samples within each experiment were processed under identical treatment conditions. For each membrane, 1–3 target proteins were detected according to molecular weight, and β‐actin from the same membrane was used for normalization.

### 2.10. Immunofluorescence Co‐Localization Analysis

EOMA cells were seeded on confocal dishes, fixed with 4% paraformaldehyde, permeabilized with Triton X‐100, and blocked with 5% BSA. Cells were then incubated overnight at 4°C with the following primary antibodies: anti‐NLRP6 (1:200), anti‐ASC (1:200), and anti‐Caspase‐1 (1:200), followed by Alexa Fluor‐488 or Alexa Fluor‐555 conjugated secondary antibody (1:200; Thermo Fisher, USA, Cat. Number A21206/A31572). Samples were counterstained with DAPI (100 µL) and imaged using a laser confocal microscope (Carl Zeiss, Germany).

### 2.11. Statistical Analysis

All data in this experiment were derived from three or more independent samples. Statistical analyses were performed using GraphPad Prism 10 software. Data normality and homogeneity of variances were assessed prior to statistical analysis. Quantitative data are presented as mean ± standard deviation (x̄ ± s). One‐way ANOVA was used for multiple‐group comparisons, and an independent samples *t*‐test was used for two‐group comparisons.

## 3. Result

### 3.1. scRNA‐seq Identifies Nine Cell Types and Five Endothelial Cell Subgroups in Healthy Dental Pulp

After quality control and batch effect correction, a total of 31,592 cells were obtained (Figure 1A). Based on the list of characteristic genes for cell types (Table S1), dimensionality reduction and clustering analysis were performed, resulting in the identification of nine distinct cell clusters: fibroblasts (14,039), ECs (5453), bone marrow mesenchymal stem cells (4830), T cells (2324), glial cells (3071), pulp cells (1173), B cells (279), epithelial cells (265), and osteoblasts (158) (Figure 1B, C). This indicates that fibroblasts comprise the majority of cells in healthy dental pulp, while ECs rank second, constituting approximately one‐fifth of the total cell population. Utilizing the EC subgroup markers (Table S2), the ECs were further classified into five categories. The relative proportions of EC subtypes in the dental pulp were as follows: lymphatic ECs > arterial ECs > venous ECs > capillary ECs > CD34 + ECs (Figure 1D, E). These findings highlight the heterogeneity of dental pulp ECs and provide a basis for investigating the functional relevance of endothelial subpopulations in inflammatory responses.

Figure 1Cellular composition and endothelial cell subtype characterization in healthy dental pulp. (A) UMAP visualization of healthy dental pulp single‐cell RNA sequencing (scRNA‐seq) data before and after batch effect correction. (B) UMAP plot showing unsupervised clustering of major cell populations in healthy dental pulp based on scRNA‐seq. (C) Feature plots displaying the expression of representative marker genes used to identify fibroblasts and endothelial cells (ECs). (D) UMAP visualization of ECs following subclustering analysis. (E) Heatmap showing the expression patterns of marker genes defining each EC subtype.

**Figure:**
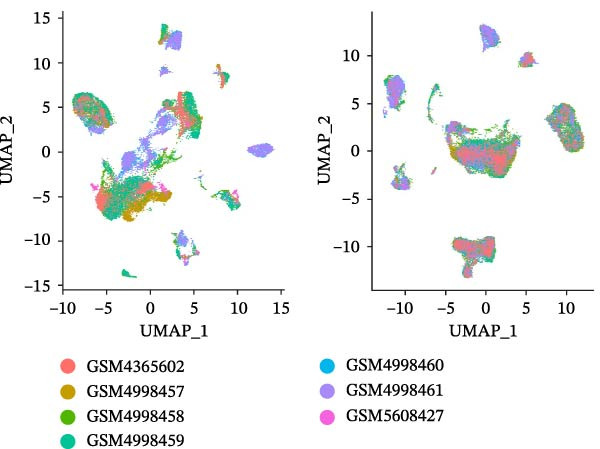
(A)

**Figure:**
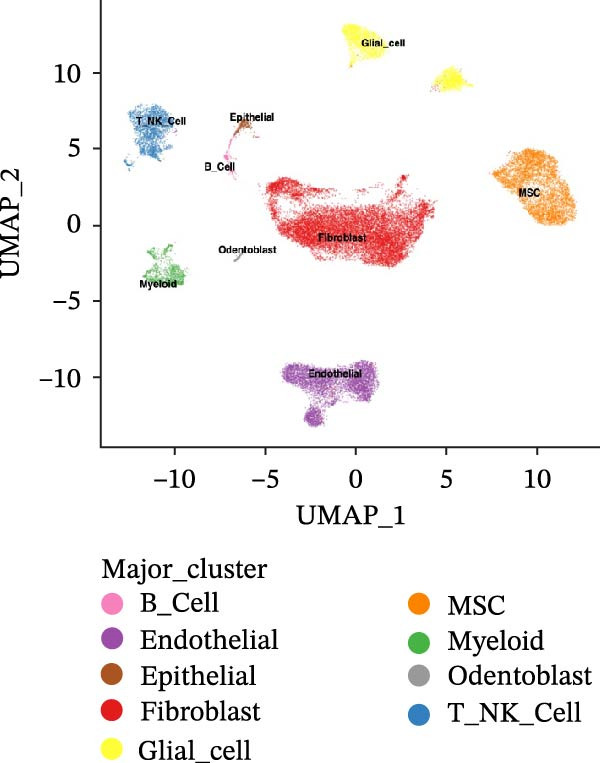
(B)

**Figure:**
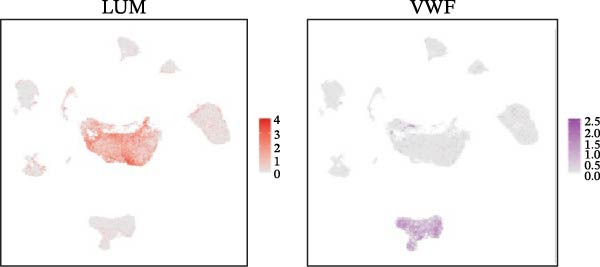
(C)

**Figure:**
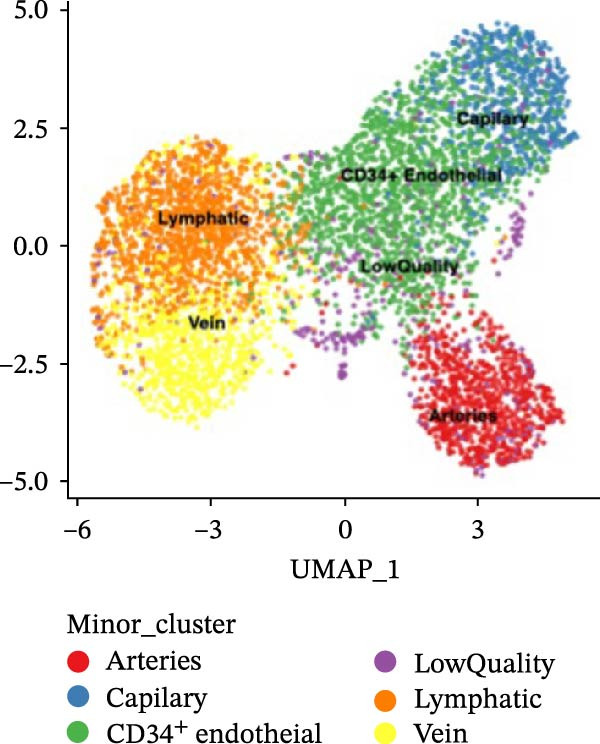
(D)

**Figure:**
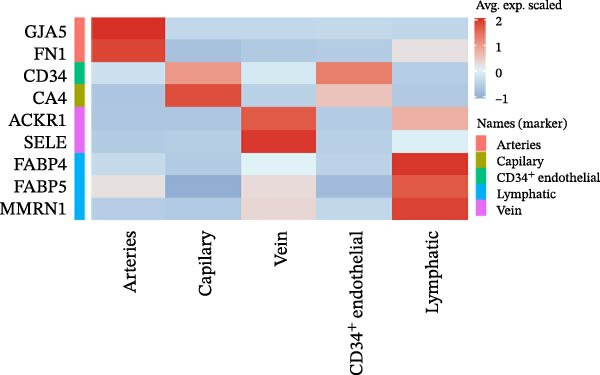
(E)

### 3.2. Pyroptosis is Associated With Pulpitis and Regulated by EZH2

Based on GSE77459, analysis of the pulpitis DEGs using a volcano plot revealed that a total of 25,137 genes exhibited expression changes between healthy pulp tissue and pulpitis tissue. Among these, 189 genes showed significant differences (log2|FC| ≥ 2, *P*_adj_ < 0.05), with 180 upregulated and 9 downregulated genes (Figure 2A). GSEA indicated a significant enrichment of pyroptosis‐related signatures in pulpitis, suggesting a positive association (*p*  < 0.01). Examination of 33 PRGs further confirmed significant enrichment in pulpitis tissues compared with healthy pulp (NES > 1, *p*  < 0.01), with 19 core genes identified at the leading edge of the enrichment peak (Figure 2B). Collectively, these findings indicate that PRGs are significantly associated with pulpitis.

Figure 2Pyroptosis‐related gene expression analyses in pulpitis and EZH2 knockout dental pulp. (A) Volcano plot showing differentially expressed genes (DEGs) between healthy pulp tissue and pulpitis tissue. (B) Gene set enrichment analysis of pyroptosis‐related genes in pulpitis tissue. (C) Heatmap showing Pearson correlation between histone methylation‐related genes and DEGs in pulpitis tissue. (D) Volcano plot displaying DEGs between EZH2 conditional knockout (CKO) and wild‐type (WT) dental pulp tissues. (E) Gene ontology biological process enrichment analysis of DEGs between EZH2 CKO and WT dental pulp tissues. (F) Kyoto Encyclopedia of Genes and Genomes enrichment analysis of DEGs between EZH2 CKO and WT dental pulp tissues. (G) Heatmap showing the expression patterns of pyroptosis‐related genes in EZH2 CKO and WT dental pulp tissues.

**Figure:**
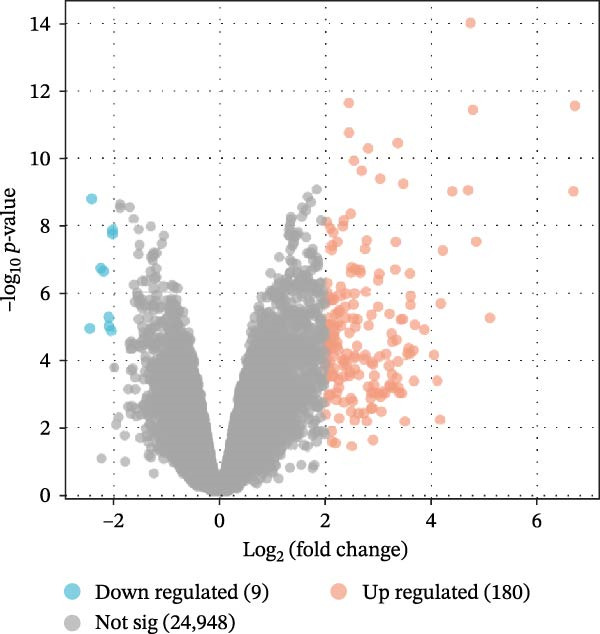
(A)

**Figure:**
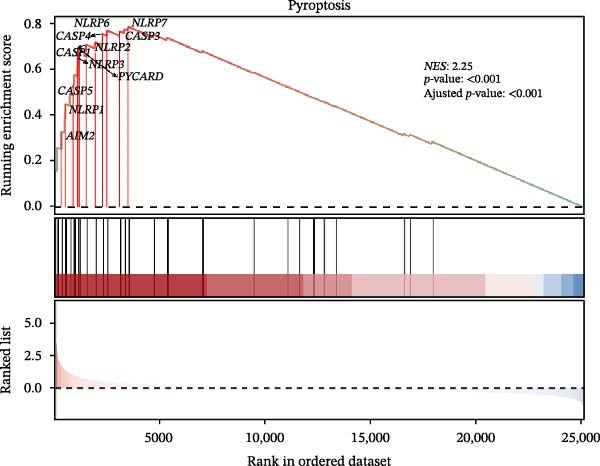
(B)

**Figure:**
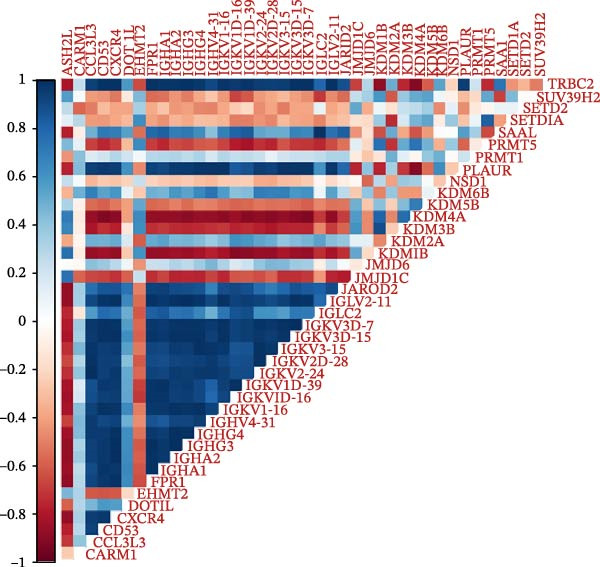
(C)

**Figure:**
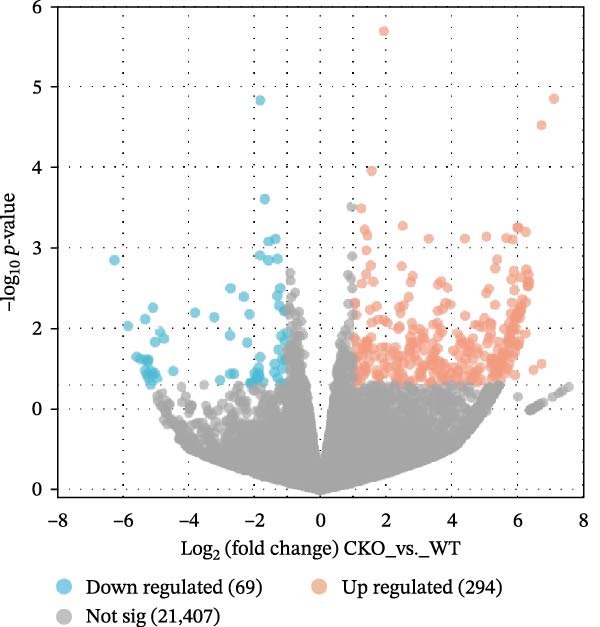
(D)

**Figure:**
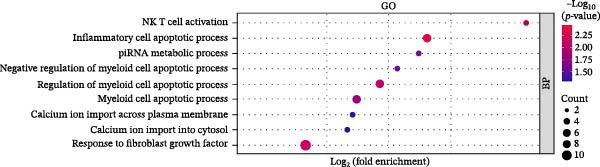
(E)

**Figure:**
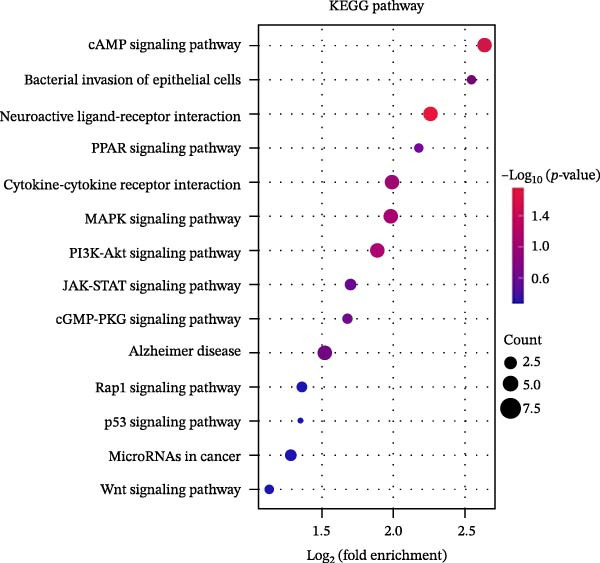
(F)

**Figure:**
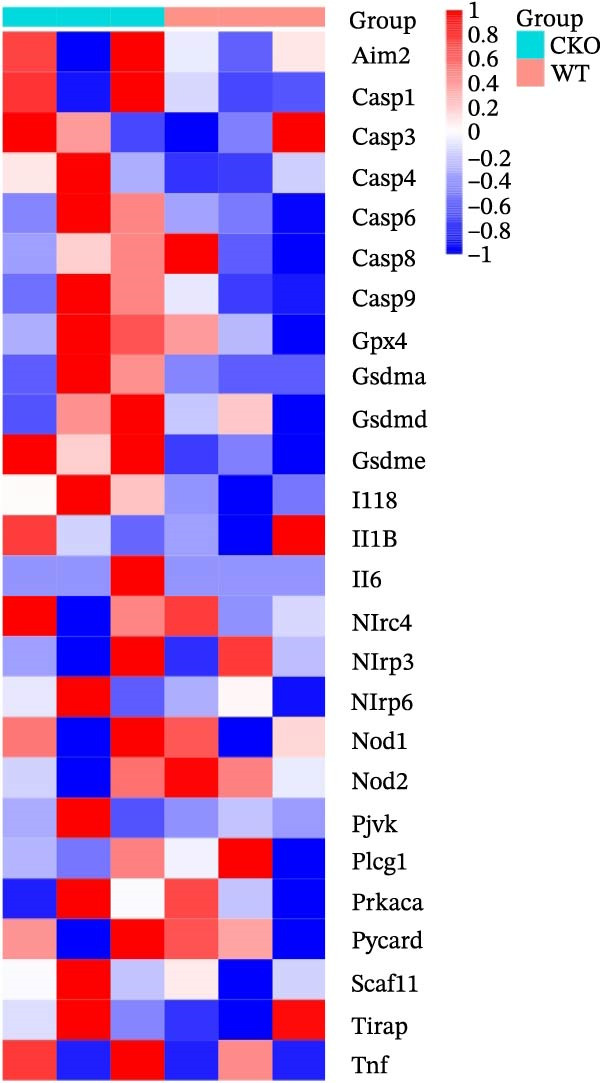
(G)

Mice displaying PCR bands corresponding to Tie2‐Cre and the floxed EZH2 allele (FIR) were identified as EZH2 CKO mice and grouped accordingly (Figure S1). To investigate potential links between histone methylation and pulpitis, correlations between histone methylation‐related genes and the primary DEGs in pulpitis were analyzed. Figure 2C shows the correlation between histone methylation‐related genes (Table S3) and the primary DEGs in pulpitis, illustrating co‐expression patterns based on Pearson correlation. Building on this observation, RNA‐seq was performed on pulp tissue from EZH2^fl/fl^;Tie2‐Cre^+/−^ mice, in which EZH2 was specifically knocked out in ECs, revealing 294 upregulated and 69 downregulated genes in the pulp tissue of EZH2^fl/fl^;Tie2‐Cre^+/−^ mice (Figure 2D). GO functional enrichment analysis showed that these DEGs were primarily involved in immune activation and cell death‐related biological processes, including apoptotic processes in myeloid and inflammatory cells and NK and T cell activation (Figure 2E). KEGG pathway analysis indicated that DEGs were mainly enriched in signaling pathways related to immunity and cell death, including cytokine–cytokine receptor interaction, MAPK signaling, and JAK‐STAT signaling (Figure 2F). These results suggest that EZH2 deficiency is associated with altered programmed cell death activity and inflammasome‐related signatures, consistent with pyroptosis in pulp tissue. Heatmap analysis revealed that EZH2 deficiency led to broad alterations in pyroptosis‐related gene expression (Table S4), including key inflammasome components and executioner genes (e.g., *Casp1*, *Gsdmd*, *Nlrp6*) (Figure 2G).

### 3.3. EZH2 is Associated With NLRP6 Inflammasome‐Related Pyroptosis in Pulpitis

FeaturePlot analysis demonstrated that key PRGs were predominantly expressed in EC subclusters, indicating an enrichment between endothelial identity and pyroptotic signaling (Figure 3A). Consistently, the PPI network revealed potential associations between EC marker genes and PRGs (Figure 3B). Pearson correlation analyses were then conducted between each PRG and *EZH2*, identifying 14 genes with an absolute Pearson correlation coefficient greater than 0.5 (Figure 3C), highlighting genes with stronger potential association. Among these, *NLRP6*, *CASP1*, and *PYCARD* were most closely related to inflammasome formation and *EZH2*, with *NLRP6* selected for further focus due to its strong relevance to oral inflammatory responses. Consistent with these findings, NLRP6 expression was markedly higher in pulpitis samples as shown by the boxplot (*p* < 0.05) (Figure 3D). Correlation analyses revealed that the Pearson coefficient between *NLRP6* and *EZH2* was 0.56 (*p* = 0.06), indicating a marginal correlation; between *CASP1* and *EZH2*, 0.60 (*p* < 0.05); and between *PYCARD* and *EZH2*, 0.69 (*p* < 0.05), both indicating significant correlations (Figure 3E–G). These findings suggest a potential role of EZH2 in regulating NLRP6‐mediated pyroptotic signaling in ECs during pulpitis.

Figure 3Expression and correlation of *EZH2* and inflammasome‐related genes in pulpitis. (A) FeaturePlot visualization of pyroptosis‐related genes across endothelial subpopulations. (B) Protein–protein interaction network of endothelial cell marker genes and pyroptosis‐related genes. (C) Pearson correlation analysis between *EZH2* and pyroptosis‐related genes. (D) Boxplot illustrating the differential expression of NLRP6 between healthy pulp tissue and pulpitis tissue. (E–G) Pearson correlation analyses between *EZH2* and (E) *NLRP6*, (F) *CASP1*, and (G) *PYCARD* ( ^∗^ *p* < 0.05).

**Figure:**
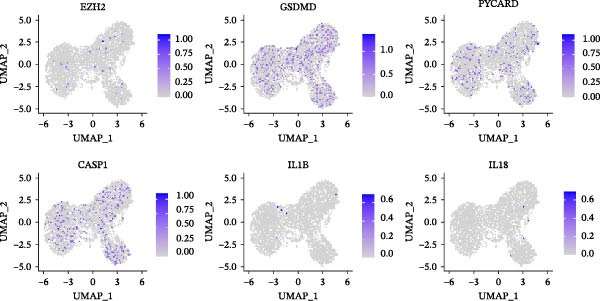
(A)

**Figure:**
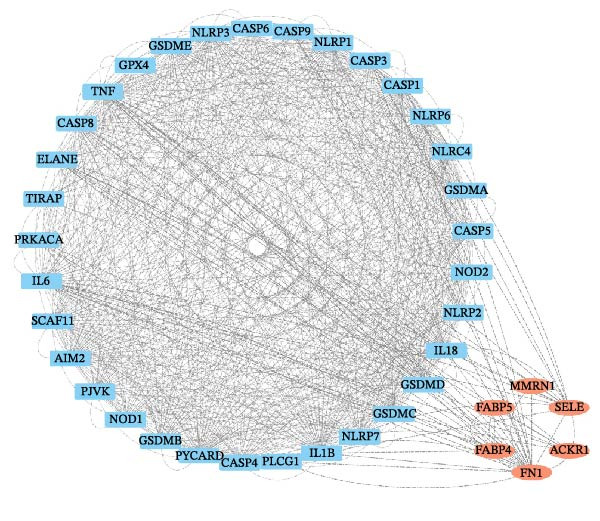
(B)

**Figure:**
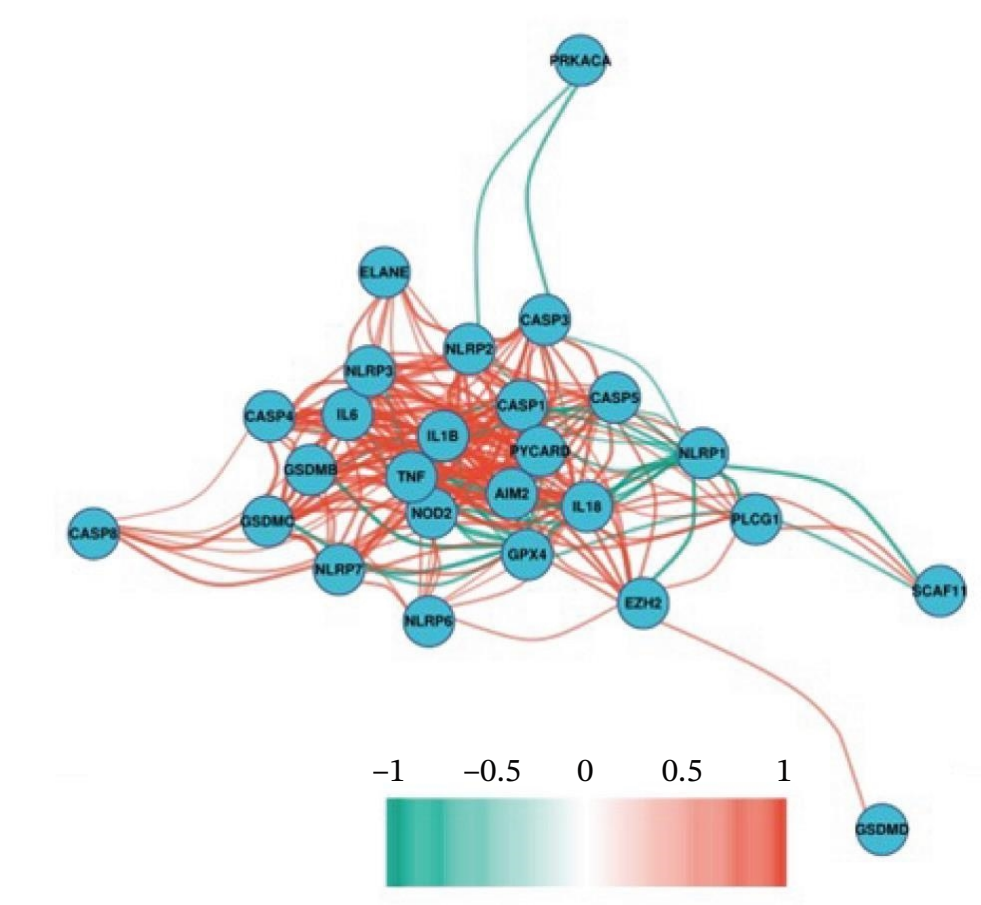
(C)

**Figure:**
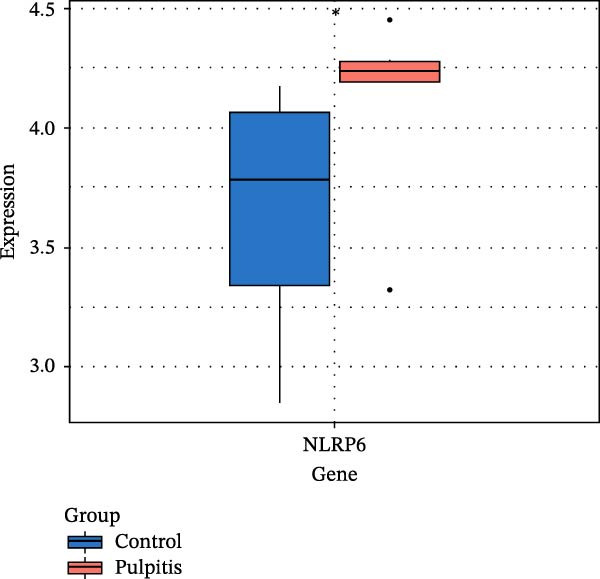
(D)

**Figure:**
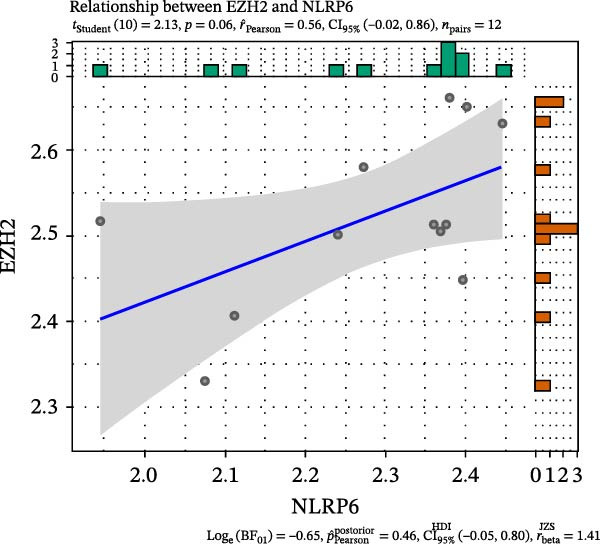
(E)

**Figure:**
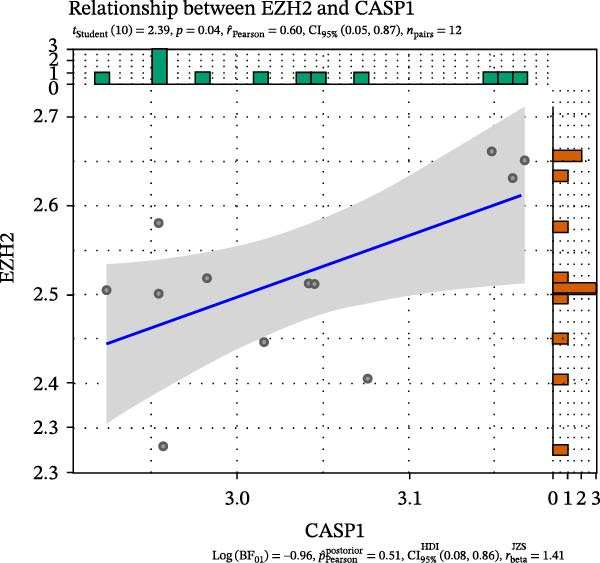
(F)

**Figure:**
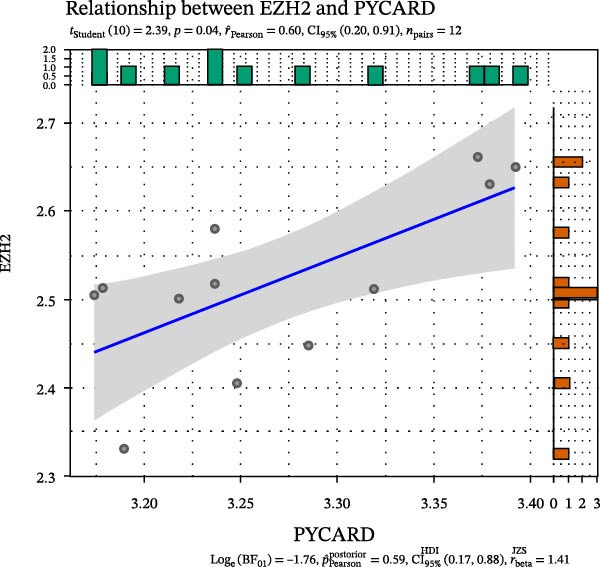
(G)

### 3.4. LPS Stimulation Upregulates EZH2 and NLRP6 Inflammasome‐Related Protein Expression in EOMA Cells

To investigate the NLRP6 inflammasome in pulpitis, EOMA cells were subjected to combined LPS and ATP stimulation, and the expression of related proteins was assessed by Western blot analysis. LPS treatment induced a dose‐dependent increase in EZH2 expression (*p* < 0.01), accompanied by upregulation of NLRP6, ASC, Pro‐Caspase‐1, Cle‐Caspase‐1, Pro‐IL‐1β, IL‐1β, and IL‐18 (*p* < 0.05) (Figure 4A–I). The elevation of Cle‐Caspase‐1 indicated cleavage of Pro‐Caspase‐1 into its active form, suggesting activation of the NLRP6 inflammasome (Figure 4E, F). Under treatment with 2 μg/mL LPS and 5 μM ATP for 24 h, the secretion of IL‐1β and IL‐18 was markedly elevated (*p* < 0.01) (Figure 4H, I), further demonstrating that inflammasome‐related proteins reached their highest expression levels under these conditions. Therefore, unless otherwise stated, all subsequent experiments employed this treatment condition.

Figure 4Inflammasome protein expression in lipopolysaccharide‐stimulated endometrial mesenchymal stromal cells. (A) Western blotting was performed to assess the expression of EZH2, NLRP6, ASC, Pro‐Caspase‐1, Cle‐Caspase‐1, Pro‐IL‐1β, IL‐1β, and IL‐18 in endometrial mesenchymal stromal cells following stimulation with different concentrations of lipopolysaccharide. β‐Actin from the same membrane was used as the loading control. (B–I) Statistical analyses of the (B) EZH2 (*n* = 4), (C) NLRP6 (*n* = 4), (D) ASC (*n* = 4), (E) Pro‐Caspase‐1 (*n* = 4), (F) Cle‐Caspase‐1 (*n* = 4), (G) Pro‐IL‐1β (*n* = 4), (H) IL‐1β (*n* = 4), and (I) IL‐18 (*n* = 4). ( ^∗^ *p* < 0.05,  ^∗∗^ *p* < 0.01,  ^∗∗∗^ *p* < 0.001,  ^∗∗∗∗^ *p* < 0.0001).

**Figure:**
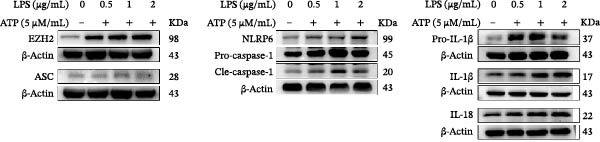
(A)

**Figure:**
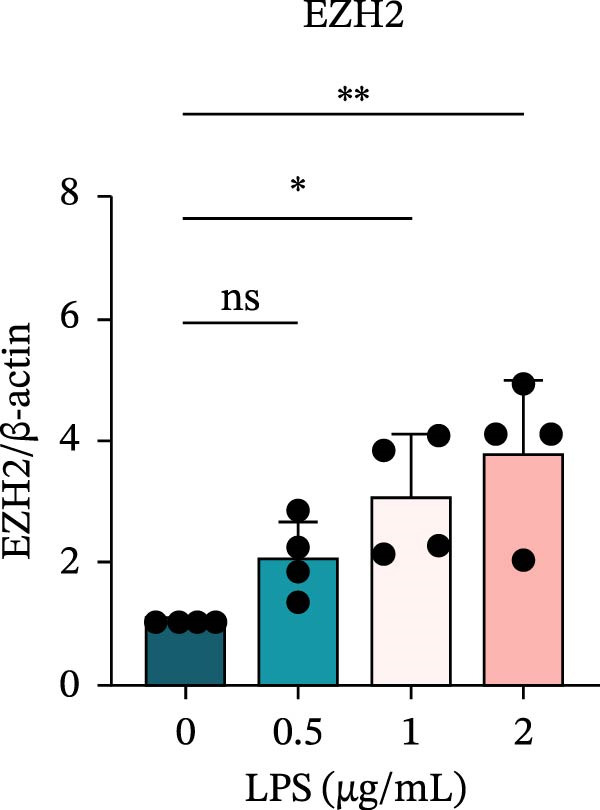
(B)

**Figure:**
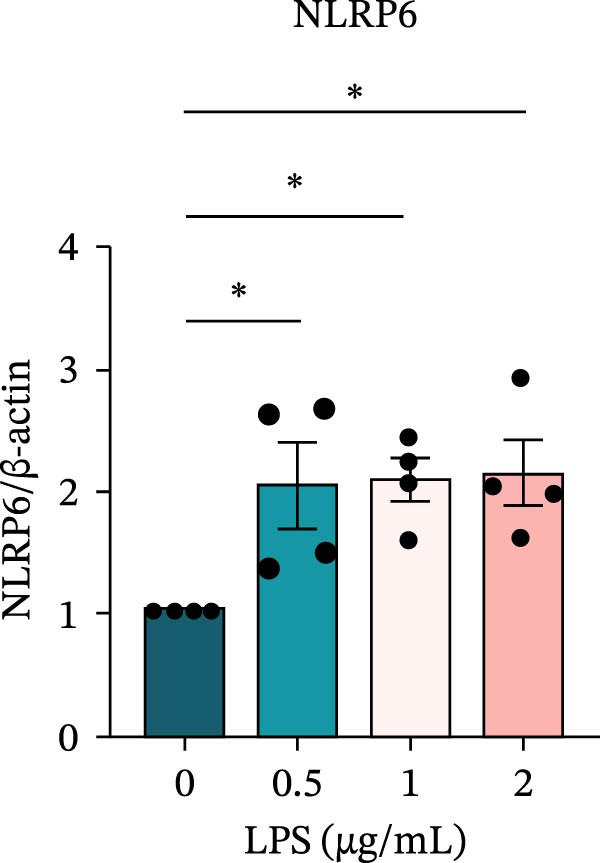
(C)

**Figure:**
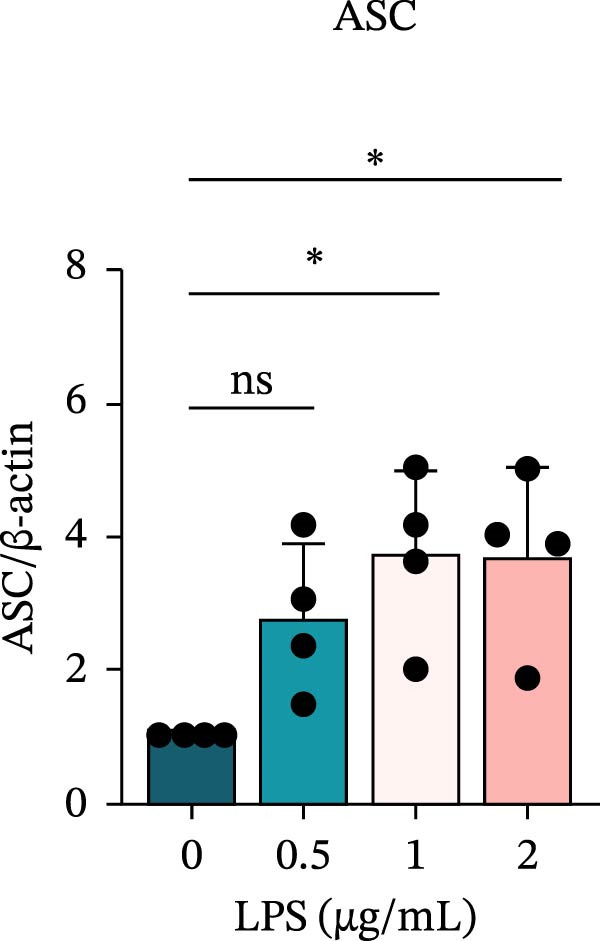
(D)

**Figure:**
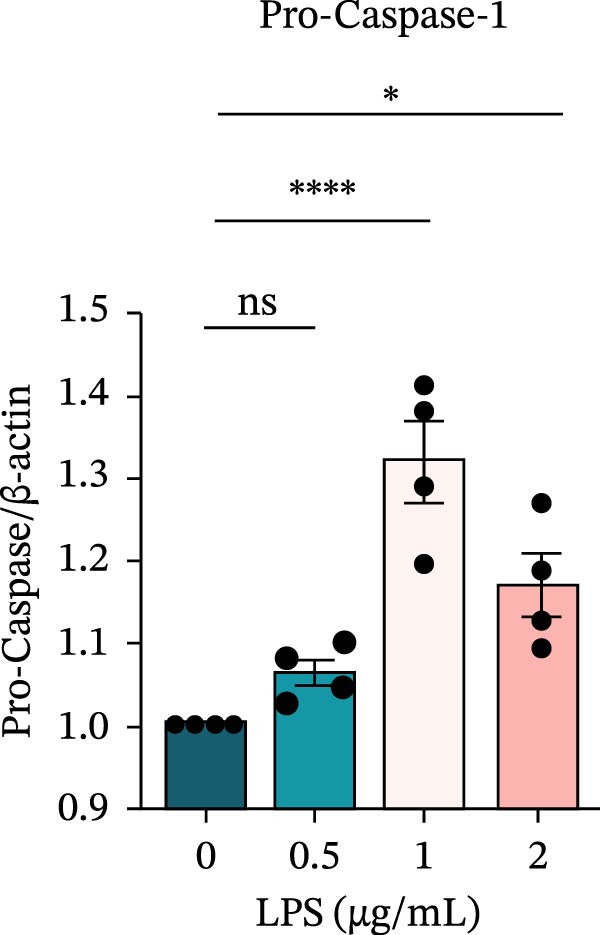
(E)

**Figure:**
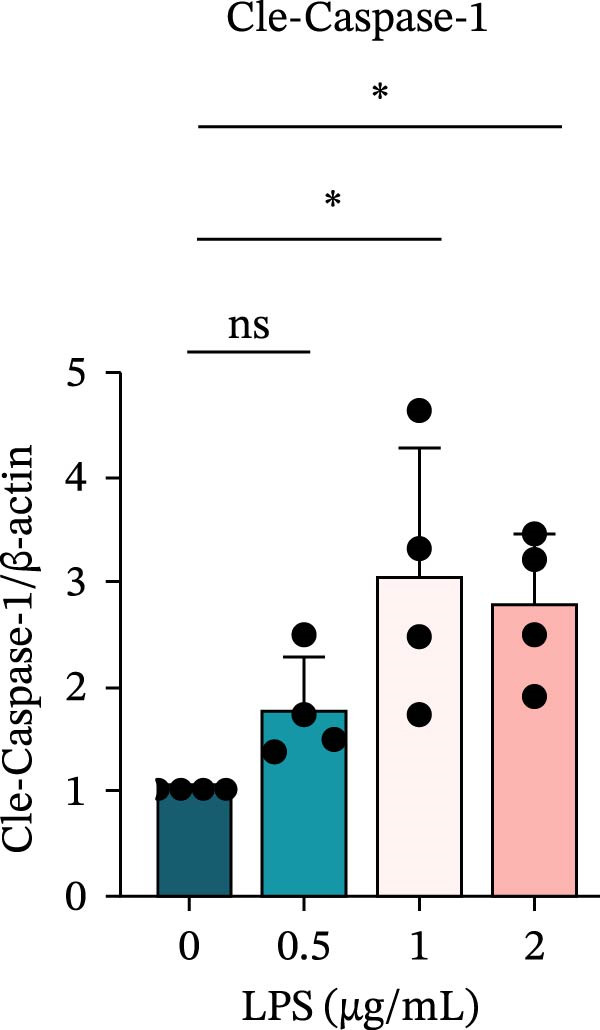
(F)

**Figure:**
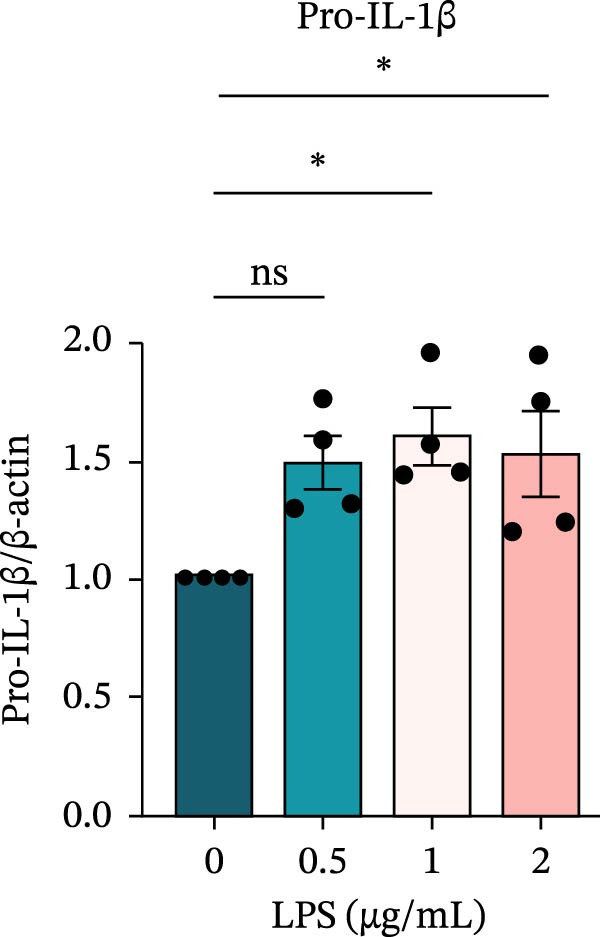
(G)

**Figure:**
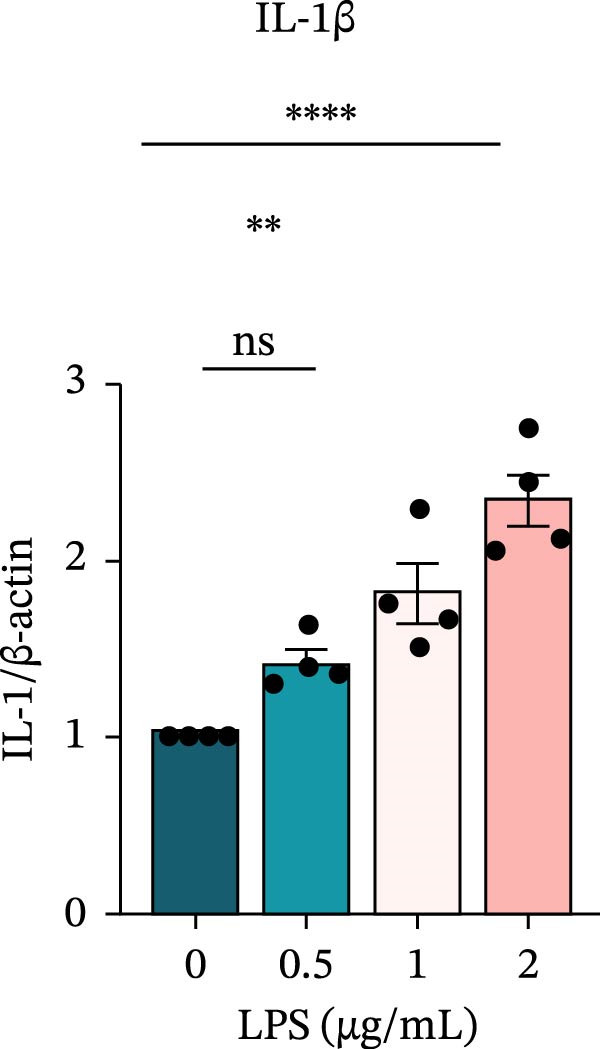
(H)

**Figure:**
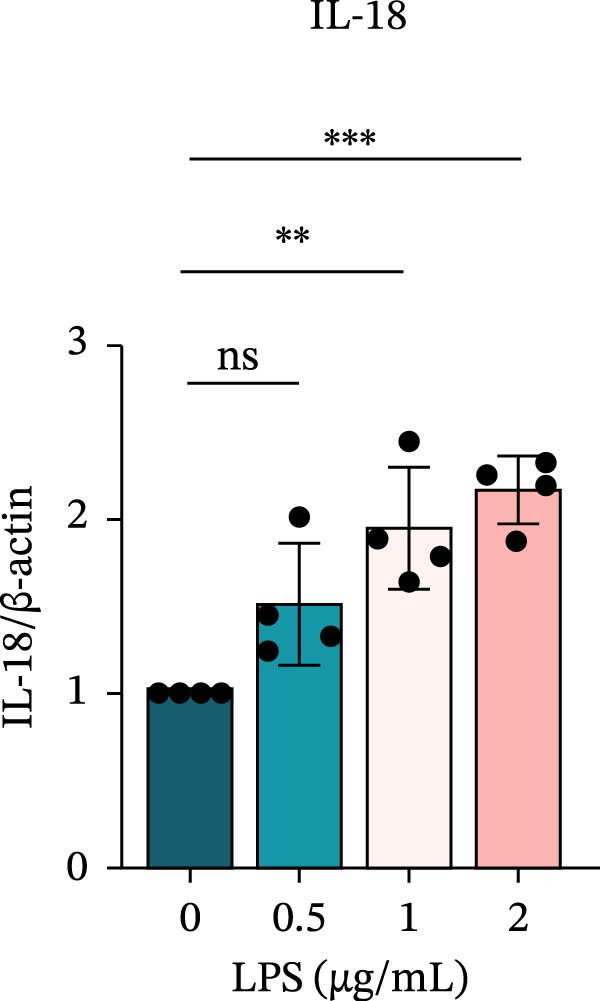
(I)

### 3.5. Confocal Microscopy Reveals NLRP6 Inflammasome Assembly in EOMA Cells

The formation of the inflammasome in EOMA cells was analyzed using confocal microscopy. Under LPS stimulation, the fluorescence signals of NLRP6 together with ASC or Caspase‐1 were markedly increased compared with the unstimulated condition, indicating LPS‐induced upregulation of these inflammasome components (Figure 5D, I). Quantitative analysis further demonstrated that the LPS‐induced increases in NLRP6 signal intensity were positively associated with those of ASC and Caspase‐1, suggesting coordinated expression of these proteins (Figure 5E, J). To further evaluate their spatial relationship under LPS stimulation, merged images were subjected to correlation analysis, which revealed high Pearson correlation coefficients exceeding 0.7 (*p* < 0.05) between NLRP6 and ASC or Caspase‐1 (Figure 5A, F). Consistently, enlarged views highlighted their close intracellular proximity (Figure 5B, G), and line‐scan analyses across selected regions showed synchronous fluorescence intensity fluctuations of NLRP6 with ASC or Caspase‐1, indicative of spatial codistribution within the same subcellular compartments (Figure 5C, H). Collectively, these findings support that LPS stimulation promotes coordinated upregulation and spatial association of NLRP6 with ASC and Caspase‐1, consistent with NLRP6 inflammasome assembly in EOMA cells.

Figure 5Colocalization analysis of NLRP6 with ASC and Caspase‐1 in lipopolysaccharide‐stimulated endometrial mesenchymal stromal cells. (A) Representative confocal microscopy image showing colocalization of NLRP6 and ASC in endometrial mesenchymal stromal (EOMA) cells following lipopolysaccharide (LPS) stimulation, supported by line‐scan fluorescence intensity profiles and Pearson correlation coefficients. (B) Magnified view highlighting local fluorescence colocalization of NLRP6 and ASC. (C) Line scan fluorescence intensity profile of NLRP6 and ASC. (D) Representative fluorescence images showing DAPI, NLRP6, ASC, and merged channels. (E) Statistical analysis of NLRP6 and ASC fluorescence intensity (*n* = 4). (F) Representative confocal microscopy image showing colocalization of NLRP6 and Caspase‐1 in EOMA cells following LPS stimulation, supported by line‐scan fluorescence intensity profiles and Pearson correlation coefficients. (G) Magnified view highlighting local fluorescence colocalization of NLRP6 and Caspase‐1. (H) Line scan fluorescence intensity profile of NLRP6 and Caspase‐1. (I) Representative fluorescence images showing DAPI, NLRP6, Caspase‐1, and merged channels. (J) Statistical analysis of NLRP6 and Caspase‐1 fluorescence intensity (*n* = 4). ( ^∗^ *p* < 0.05,  ^∗∗^ *p* < 0.01,  ^∗∗∗∗^ *p* < 0.0001).

**Figure:**
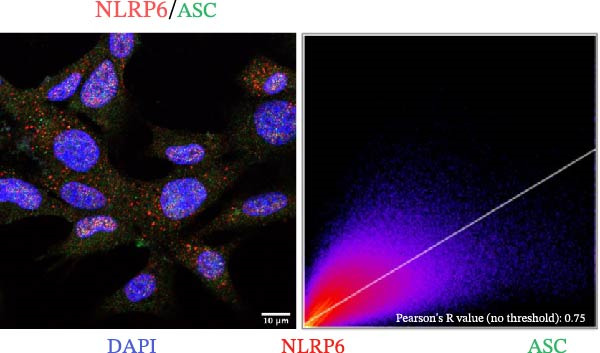
(A)

**Figure:**
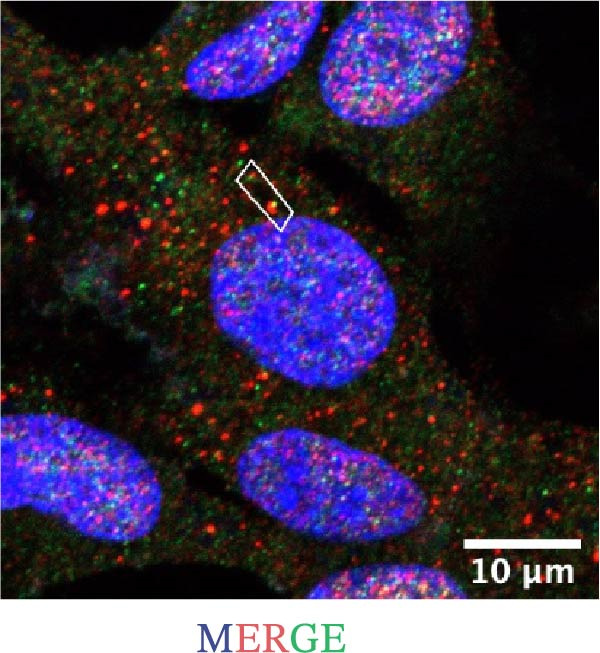
(B)

**Figure:**
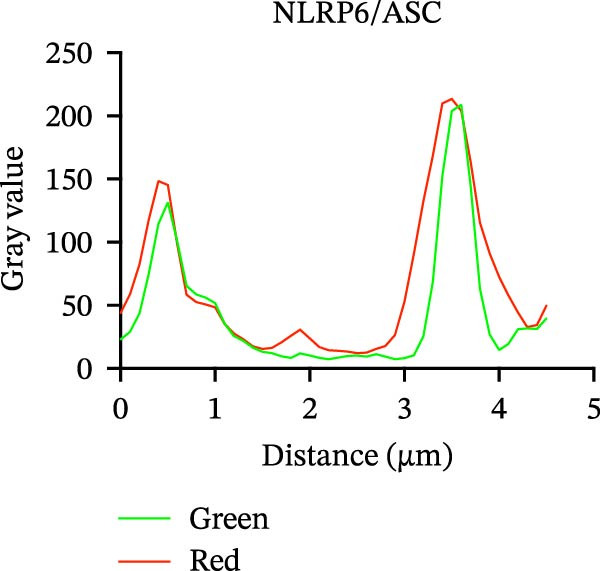
(C)

**Figure:**
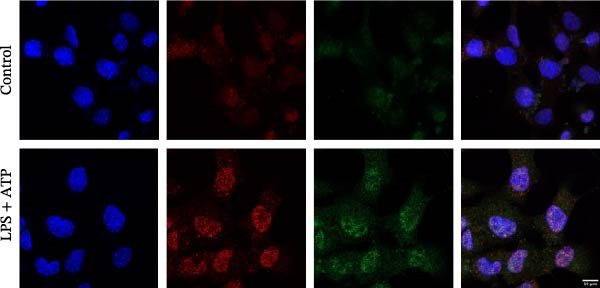
(D)

**Figure:**
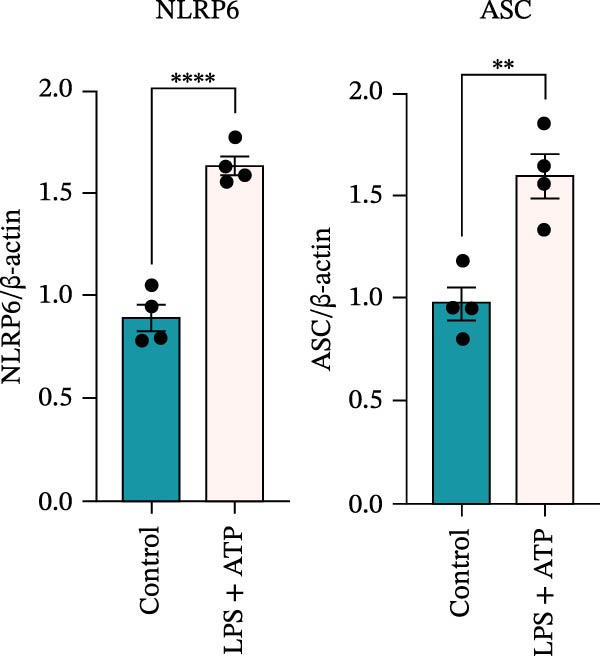
(E)

**Figure:**
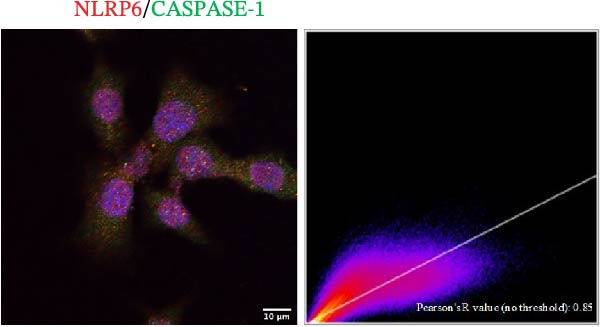
(F)

**Figure:**
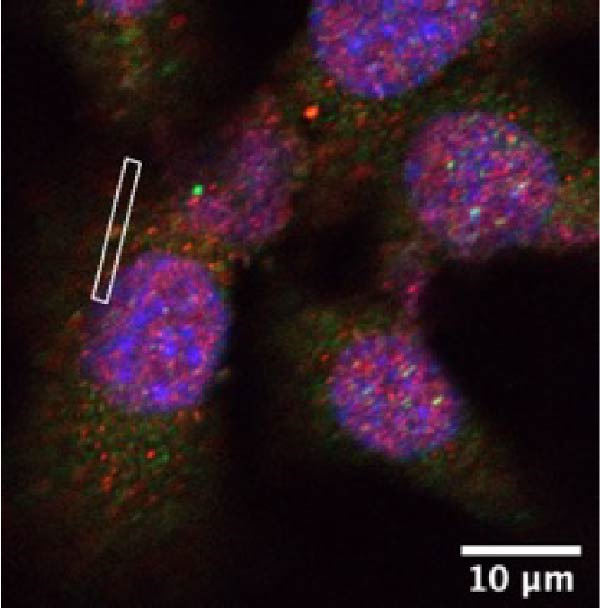
(G)

**Figure:**
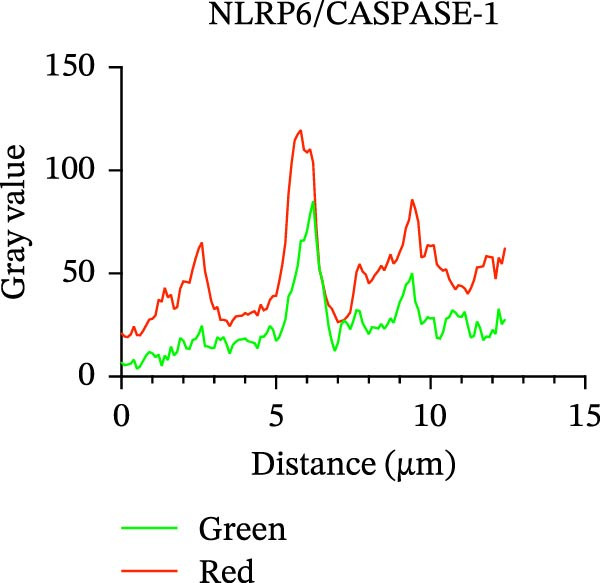
(H)

**Figure:**
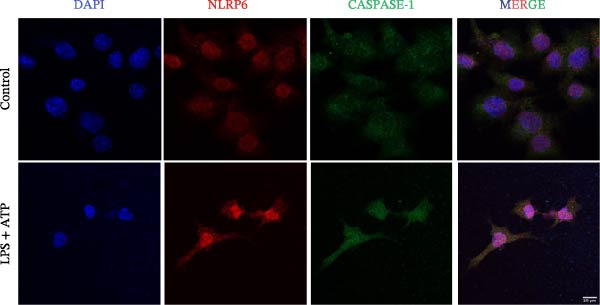
(I)

**Figure:**
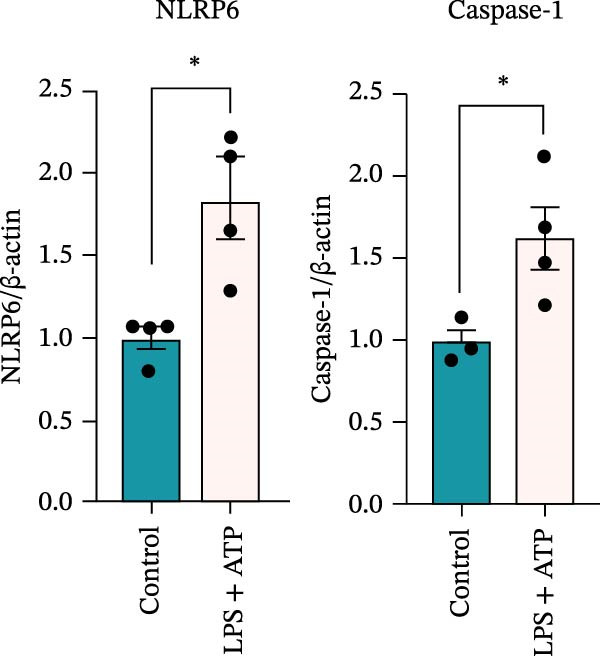
(J)

### 3.6. EZH2 Inhibition Suppresses NLRP6 Inflammasome Activation and Mitigates Pyroptosis

LPS stimulation significantly increased GSDMD‐N levels (*p* < 0.01) (Figure 6A, J, K), consistent with enhanced pyroptotic signaling. Inhibition of EZH2 by GSK126 resulted in a marked reduction in EZH2 expression (*p* < 0.05) (Figure 6A, B). The expression of inflammasome‐related proteins, including NLRP6, ASC, and Cle‐Caspase‐1, was significantly decreased (*p* < 0.01) (Figure 6A, C–F), indicating suppression of inflammasome activation. In parallel, the expression of downstream inflammatory mediators IL‐1β and IL‐18 was markedly reduced (*p* < 0.01) (Figure 6A, G–I). Moreover, the expression of the pyroptosis executor GSDMD‐N was also significantly decreased following EZH2 inhibition (*p* < 0.01) (Figure 6A, J, K). These results indicate that EZH2 inhibition attenuates NLRP6 inflammasome activation and downstream pyroptosis in LPS‐stimulated EOMA cells.

Figure 6Inflammasome and pyroptosis‐related protein expression after EZH2 inhibition. (A) Western blotting was performed to assess the expression of EZH2, NLRP6, ASC, Pro‐Caspase‐1, Cle‐Caspase‐1, Pro‐IL‐1β, IL‐1β, IL‐18, GSDMD, and GSDMD‐N in endometrial mesenchymal stromal cells following treatment with the EZH2 inhibitor GSK126. β‐actin from the same membrane was used as the loading control. (B–K) Statistical analyses of the protein expression levels of (B) EZH2 (*n* = 3), (C) NLRP6 (*n* = 3), (D) ASC (*n* = 3), (E) Pro‐Caspase‐1 (*n* = 3), (F) Cle‐Caspase‐1 (*n* = 3), (G) Pro‐IL‐1β (*n* = 3), (H) IL‐1β (*n* = 3), (I) IL‐18 (*n* = 3), (J) GSDMD (*n* = 3), and (K) GSDMD‐N (*n* = 3). ( ^∗^ *p* < 0.05,  ^∗∗^ *p* < 0.01,  ^∗∗∗^ *p* < 0.001,  ^∗∗∗∗^ *p* < 0.0001).

**Figure:**
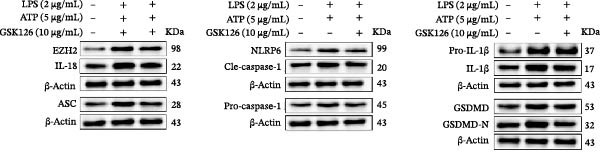
(A)

**Figure:**
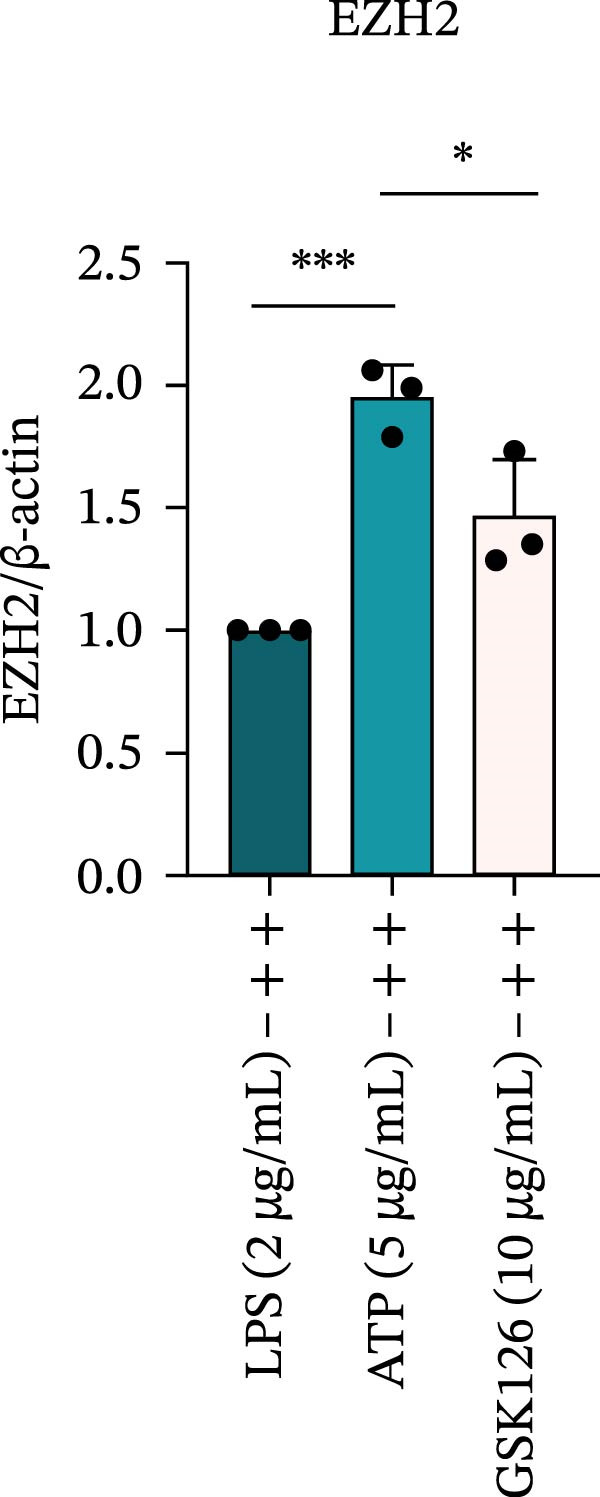
(B)

**Figure:**
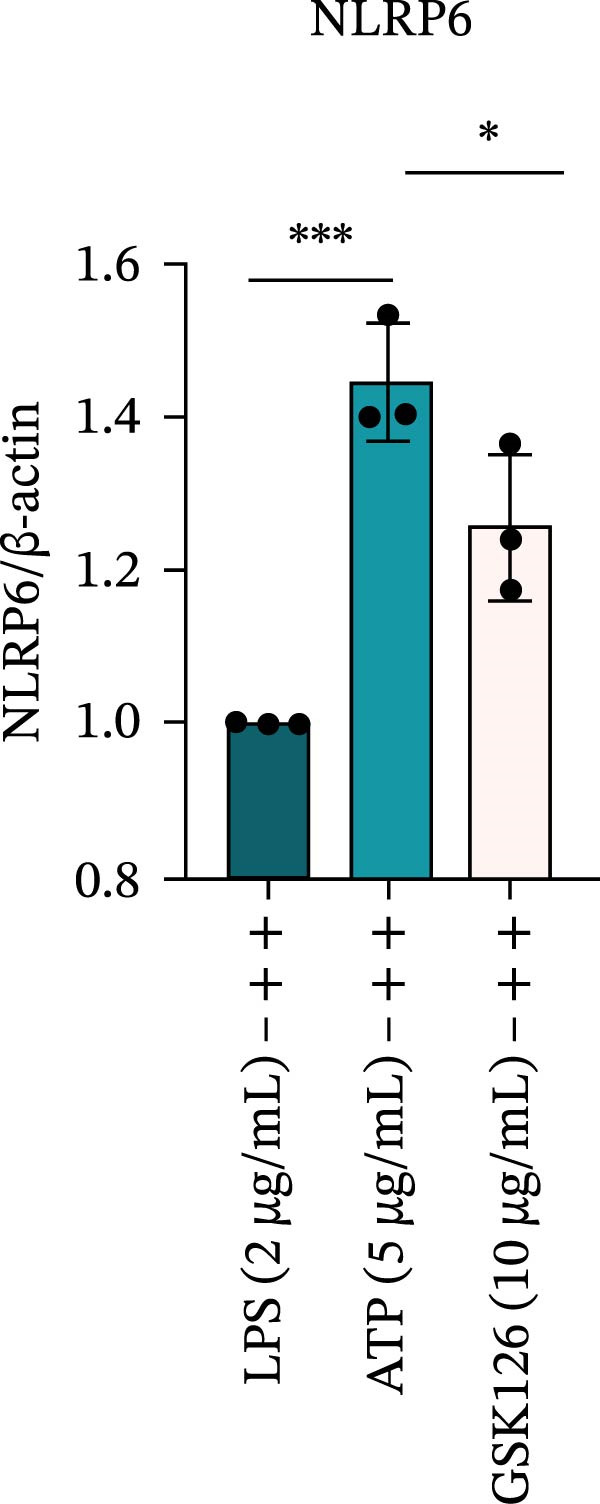
(C)

**Figure:**
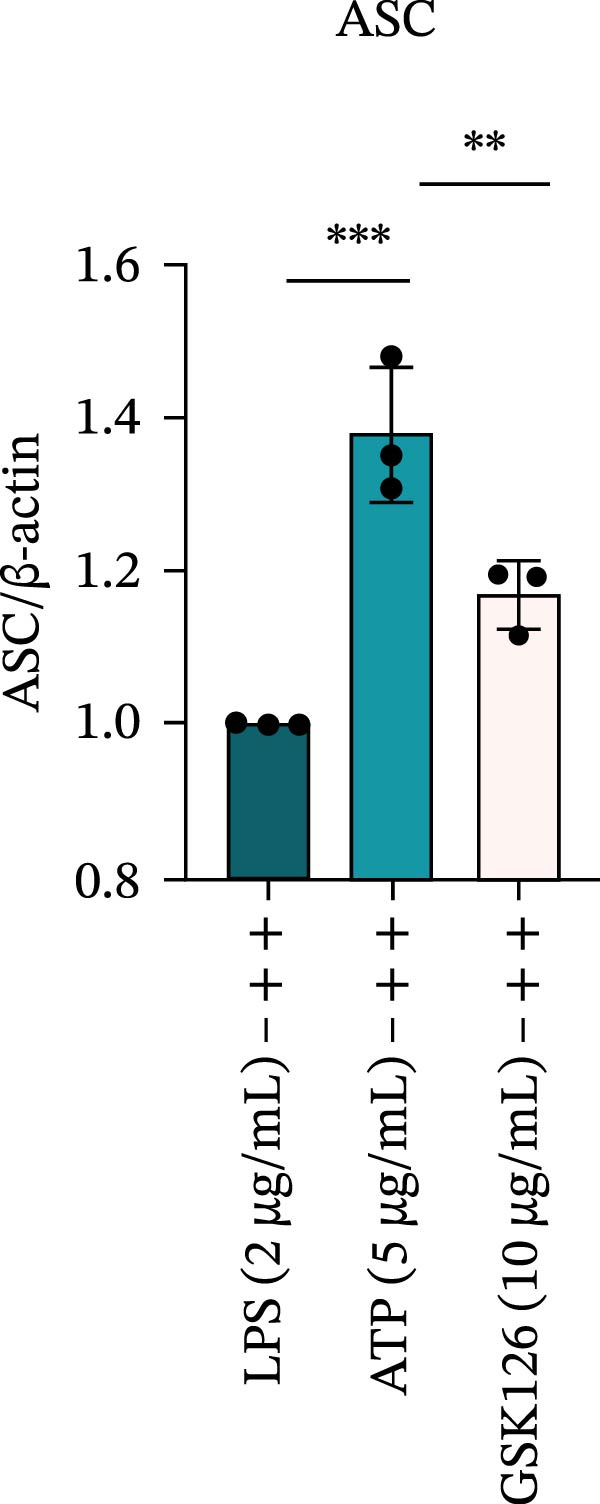
(D)

**Figure:**
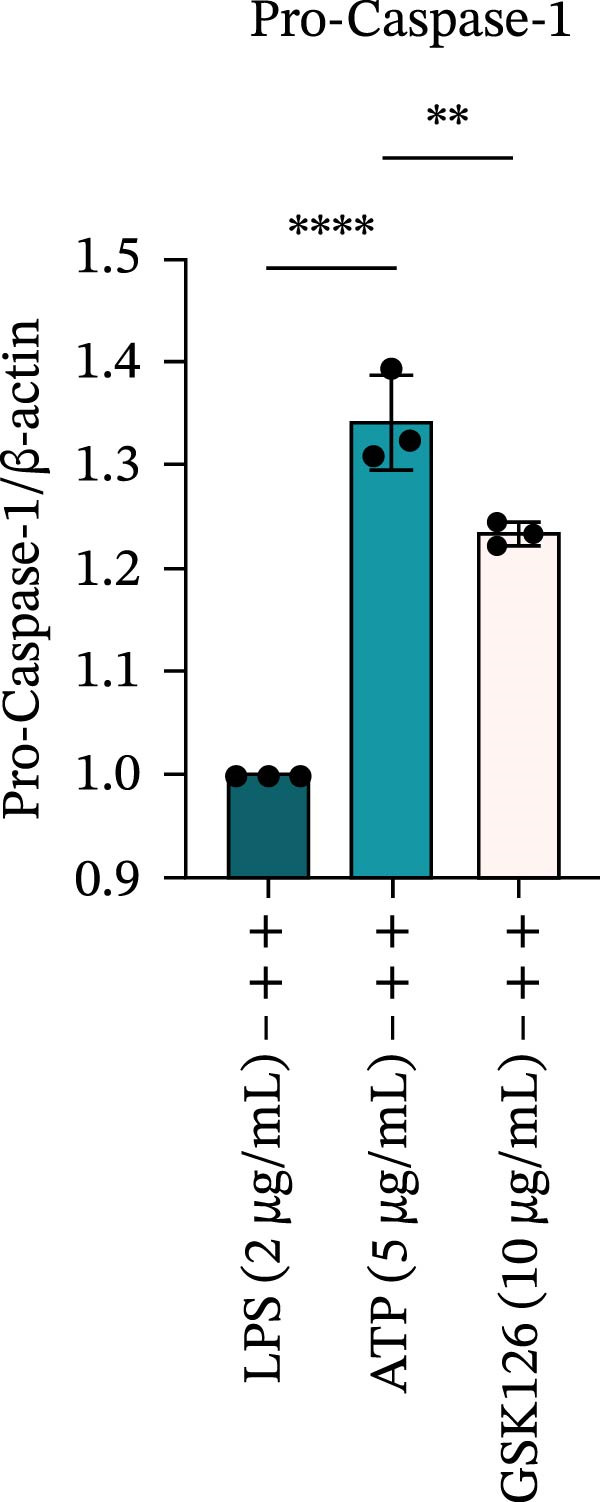
(E)

**Figure:**
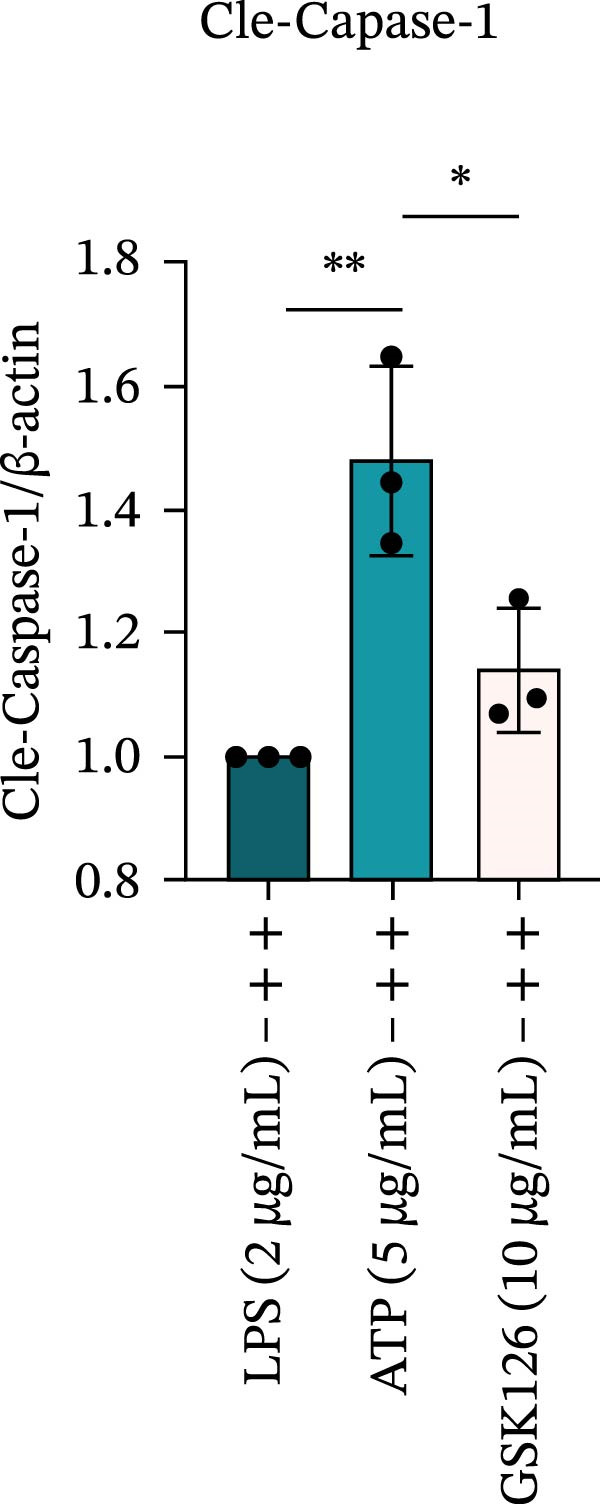
(F)

**Figure:**
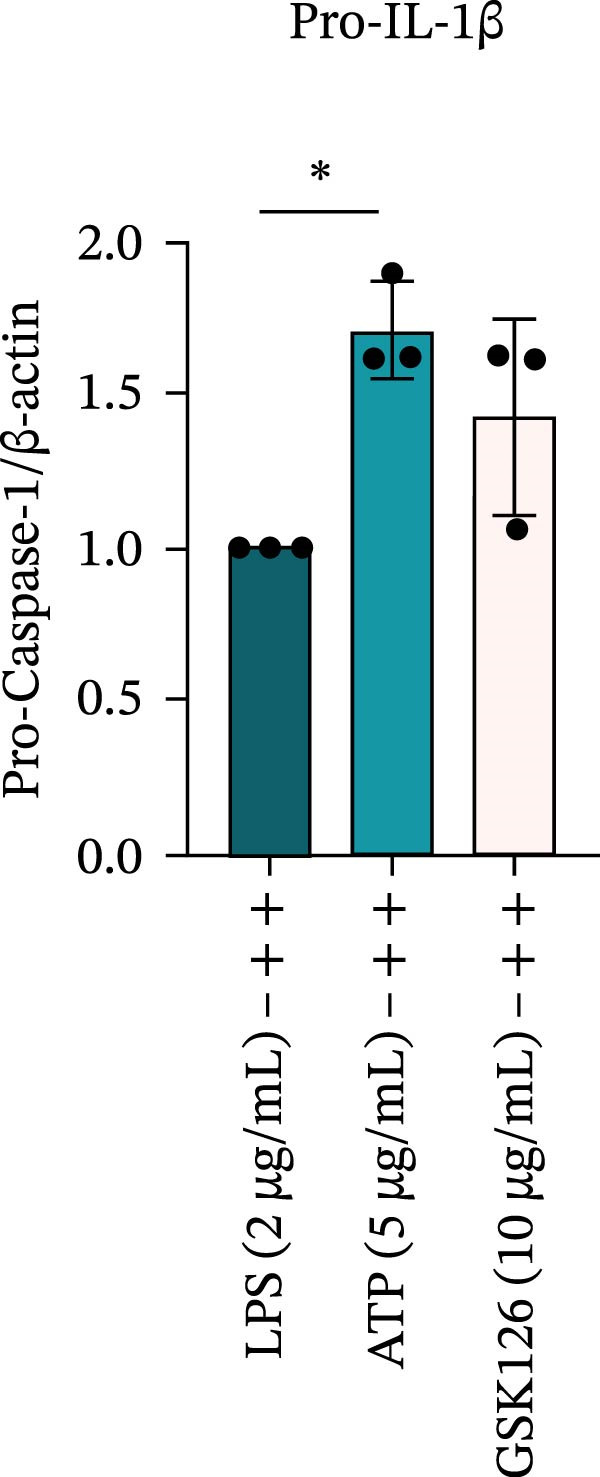
(G)

**Figure:**
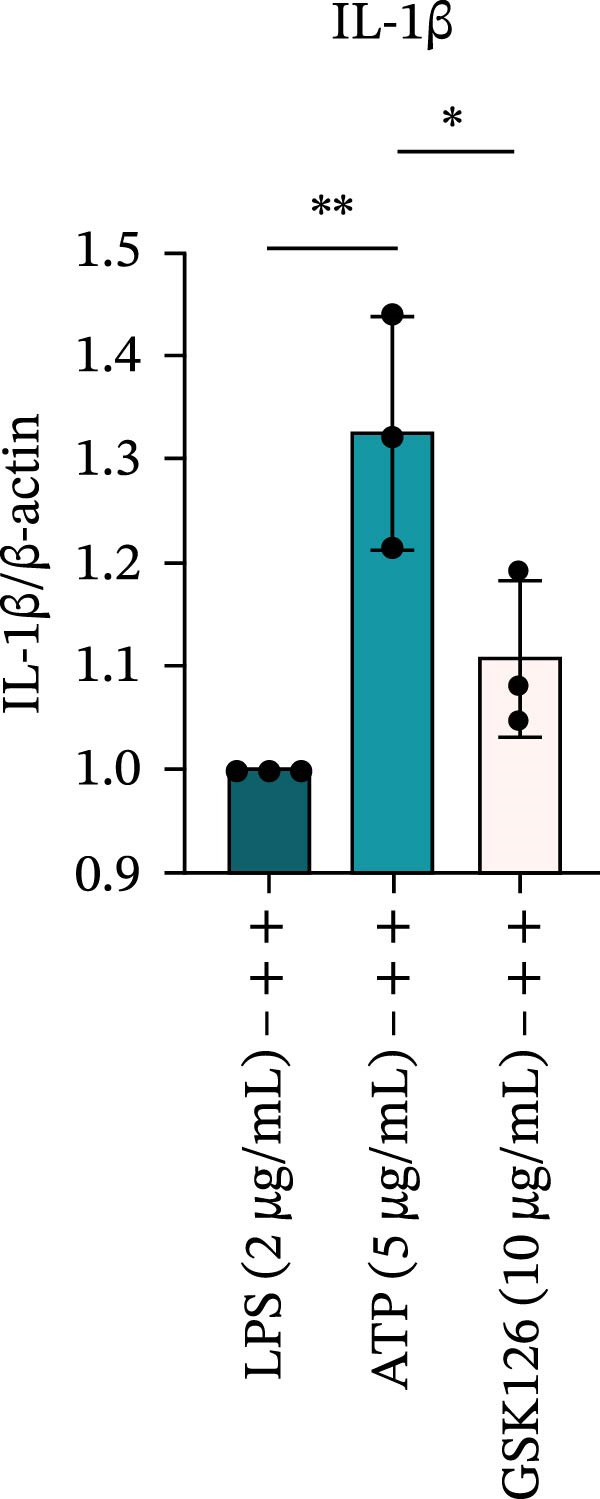
(H)

**Figure:**
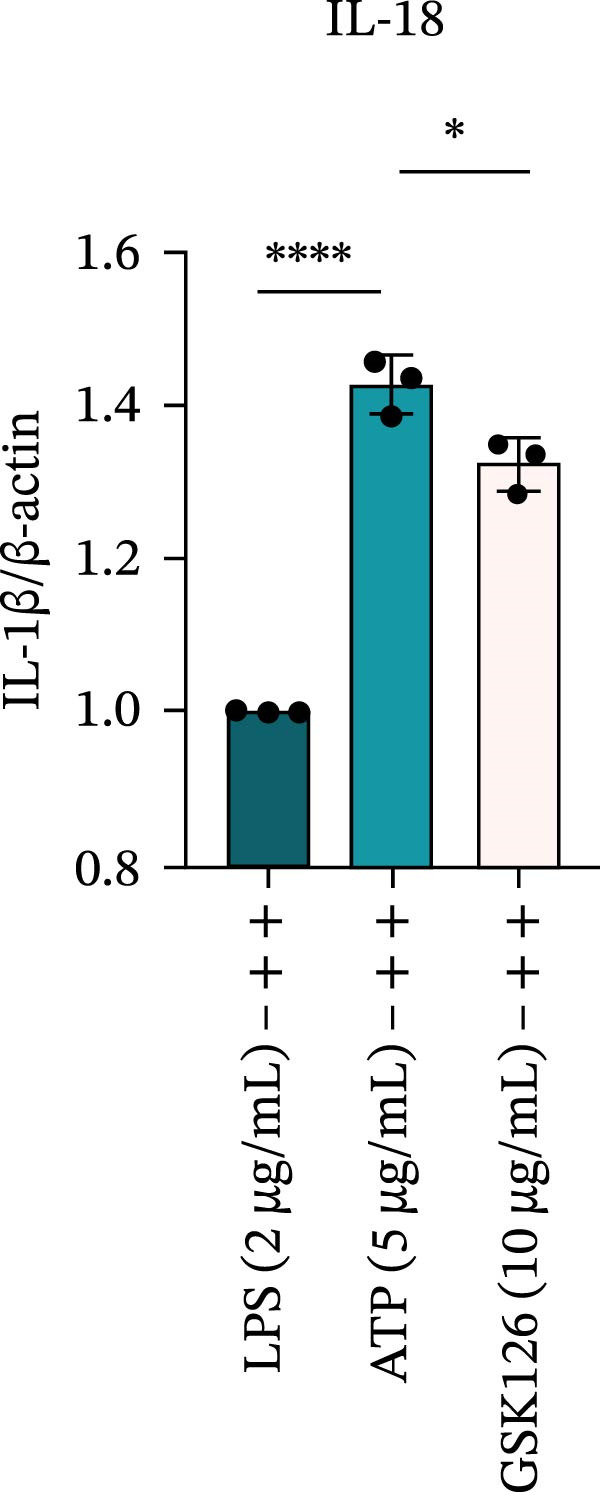
(I)

**Figure:**
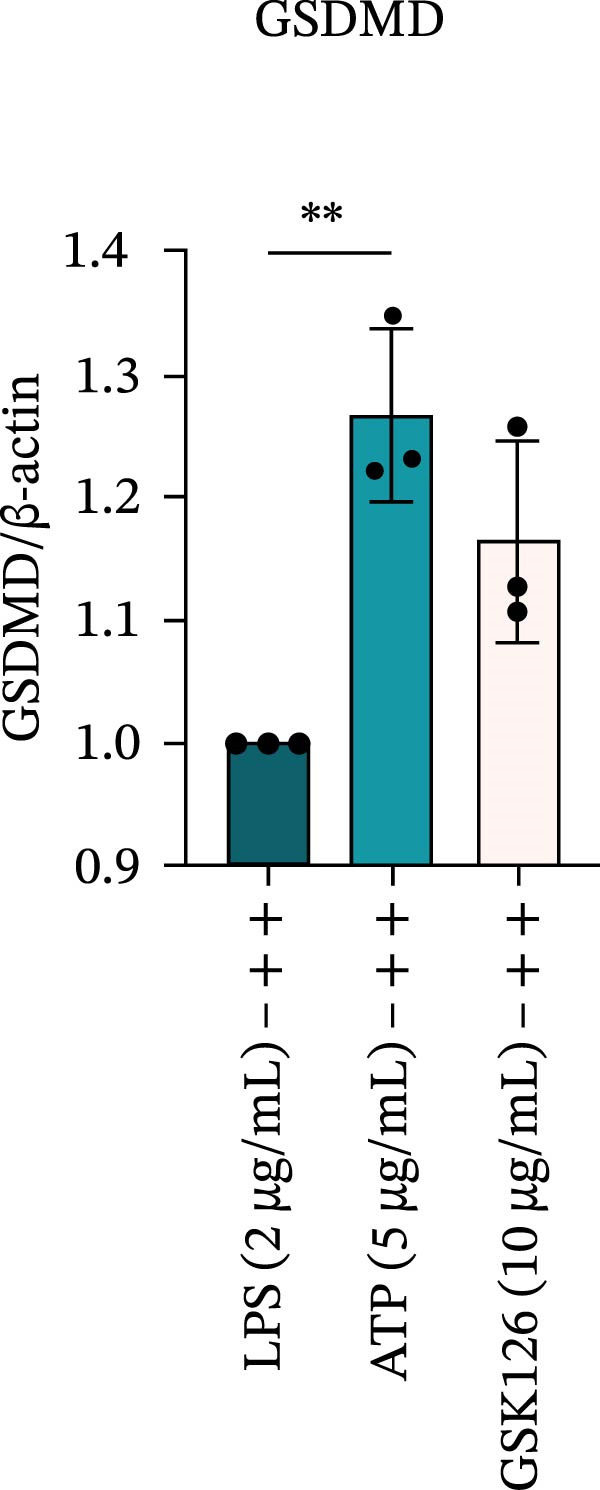
(J)

**Figure:**
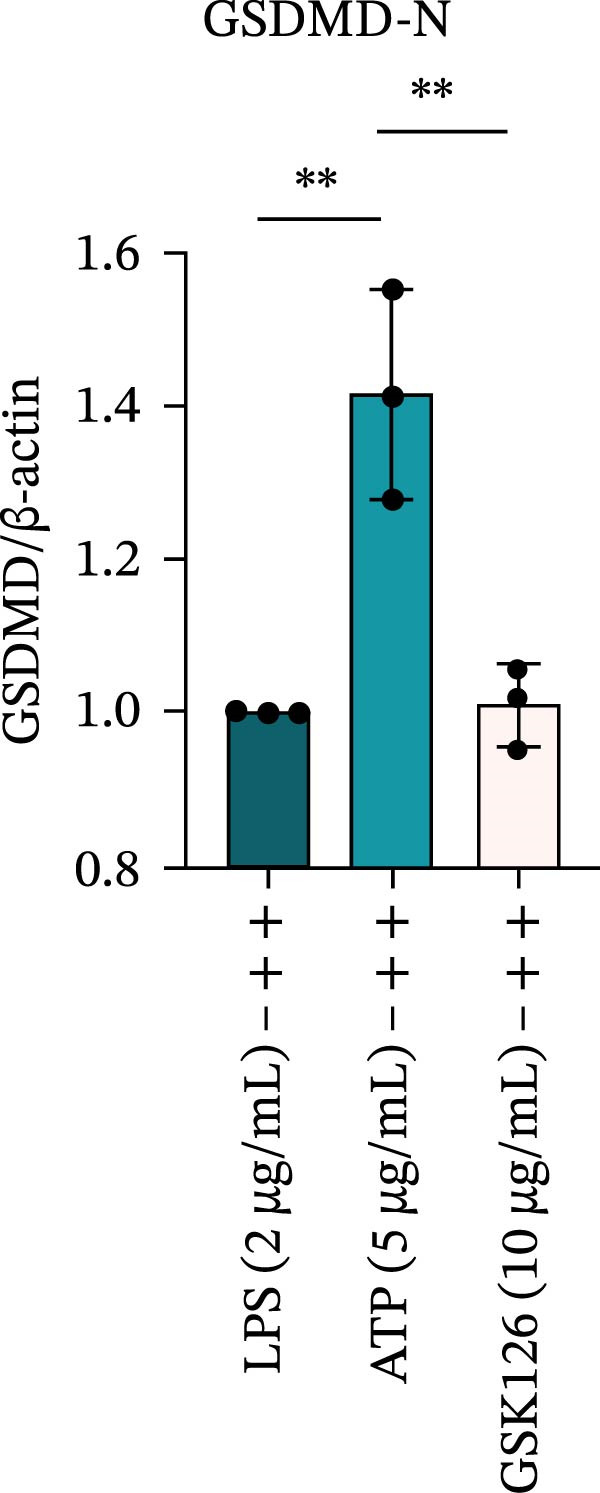
(K)

### 3.7. NLRP6 is Required for Inflammasome‐Mediated Pyroptosis

Knockdown of NLRP6 via shRNA lentiviral transduction under LPS stimulation significantly reduced the expression of inflammasome assembly proteins, including ASC, Pro‐Caspase‐1, and Cle‐Caspase‐1 (*p* < 0.01) (Figure 7A–E). Furthermore, the levels of downstream inflammatory cytokines, such as Pro‐IL‐1β, IL‐1β, and IL‐18, and the pyroptosis executioner proteins GSDMD and GSDMD‐N, were also markedly decreased (*p* < 0.05) (Figure 7A, F–J). These findings indicate that NLRP6 is essential for inflammasome assembly, activation, and the maturation of downstream cytokines, supporting a central role for NLRP6 in inflammasome‐mediated pyroptosis.

Figure 7Inflammasome and pyroptosis‐related protein expression following NLRP6 knockdown. (A) Western blotting was performed to assess the expression of NLRP6, ASC, Pro‐Caspase‐1, Cle‐Caspase‐1, Pro‐IL‐1β, IL‐1β, IL‐18, GSDMD, and GSDMD‐N in endometrial mesenchymal stromal cells following NLRP6 knockdown mediated by shRNA. β‐actin from the same membrane was used as the loading control. (B–J) Statistical analyses of the protein expression levels of (B) NLRP6 (*n* = 3), (C) ASC (*n* = 3), (D) Pro‐Caspase‐1 (*n* = 3), (E) Cle‐Caspase‐1 (n = 3), (F) Pro‐IL‐1β (*n* = 3), (G) IL‐1β (*n* = 3), (H) IL‐18 (*n* = 3), (I) GSDMD (*n* = 3), and (J) GSDMD‐N (*n* = 3). ( ^∗^ *p* < 0.05,  ^∗∗^ *p* < 0.01,  ^∗∗∗^ *p* < 0.001).

**Figure:**
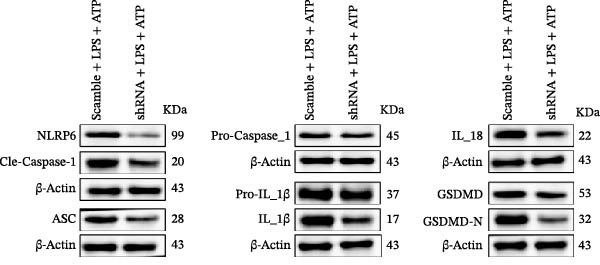
(A)

**Figure:**
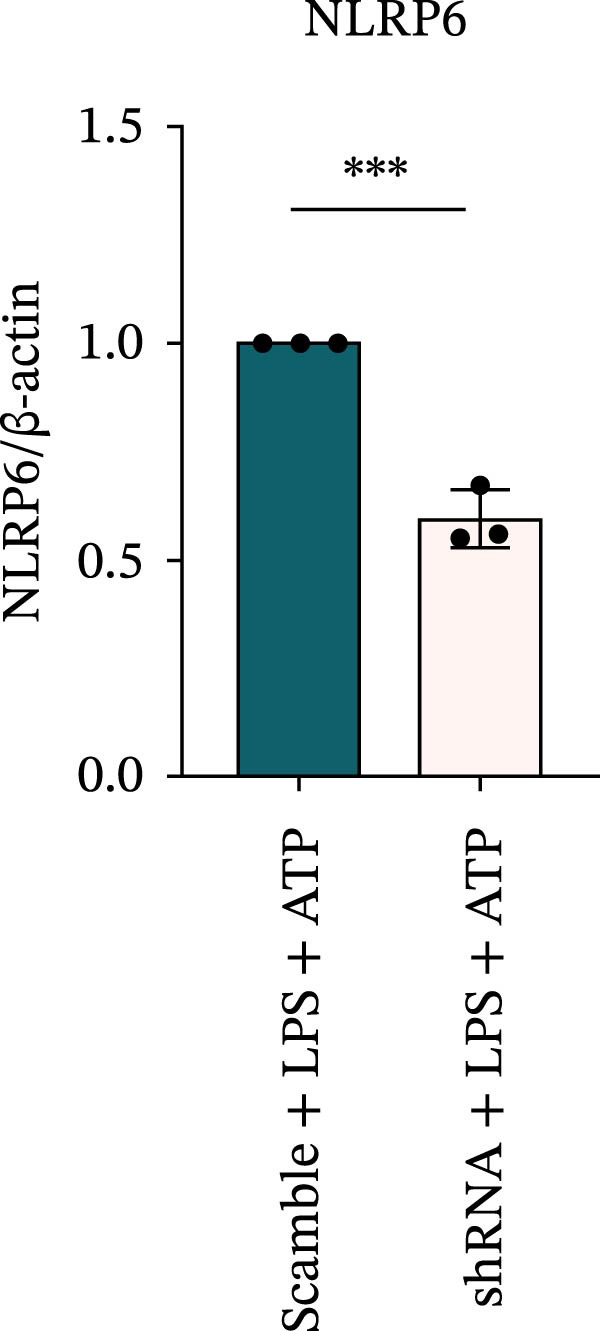
(B)

**Figure:**
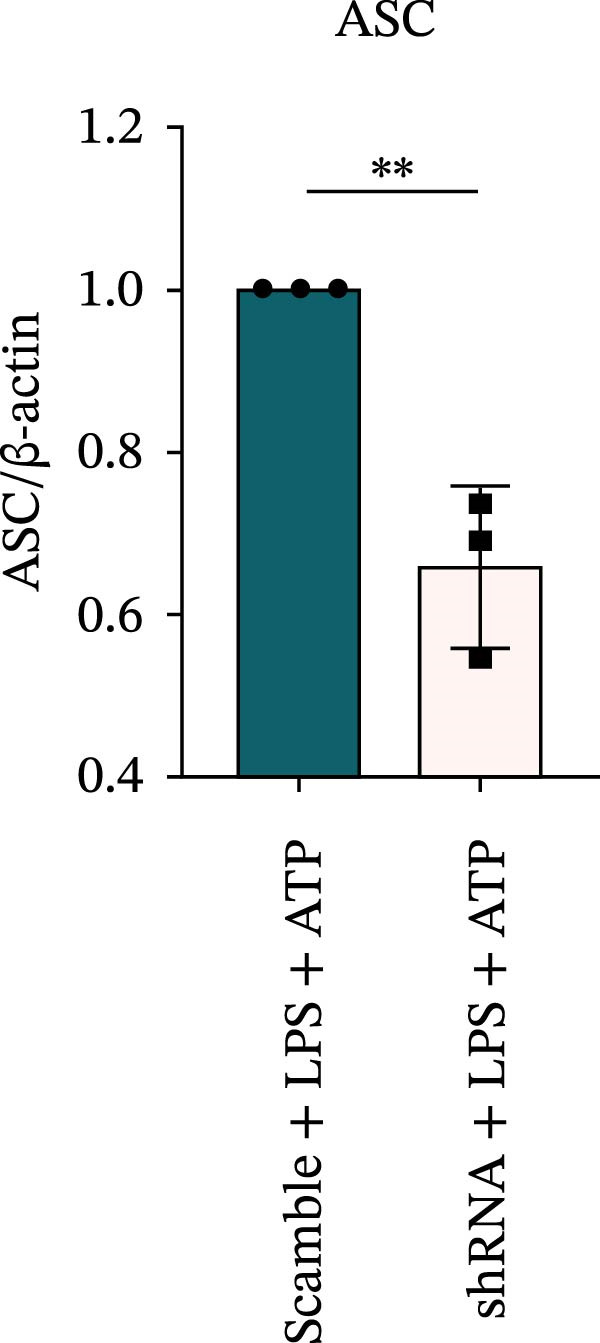
(C)

**Figure:**
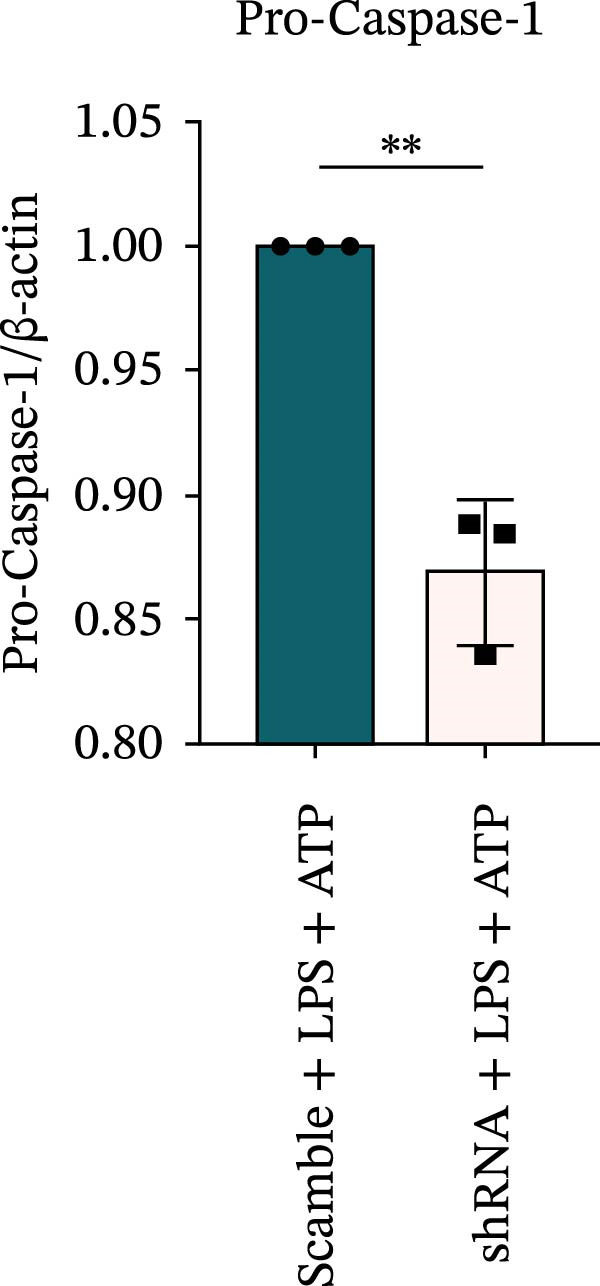
(D)

**Figure:**
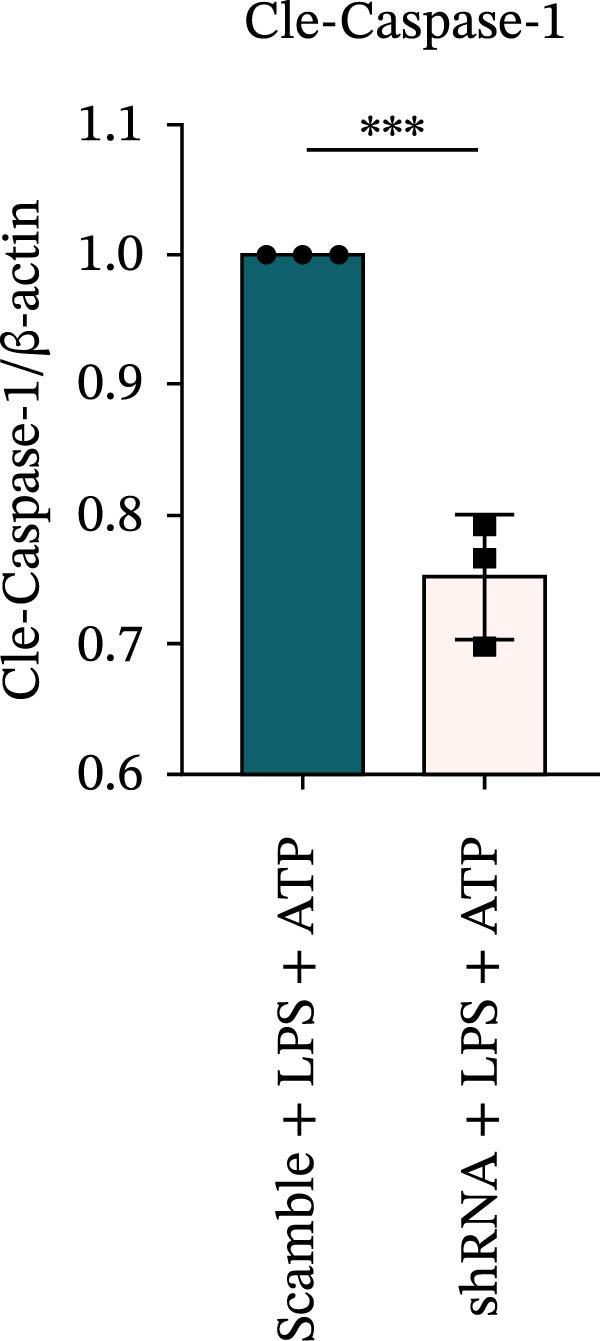
(E)

**Figure:**
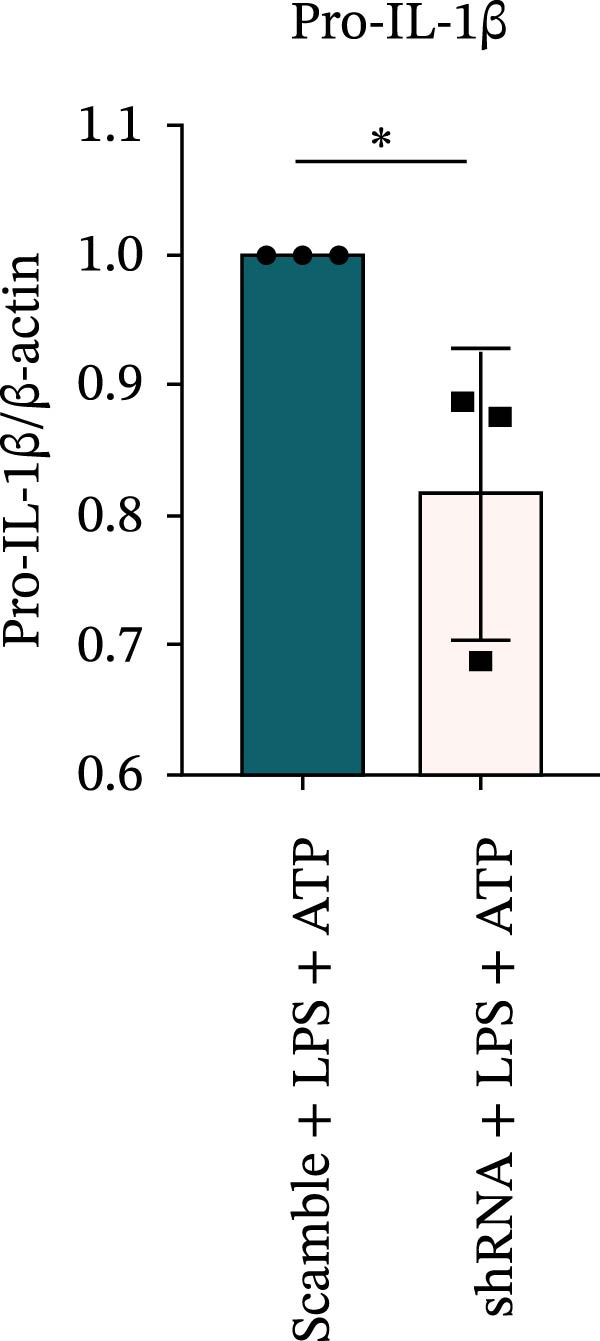
(F)

**Figure:**
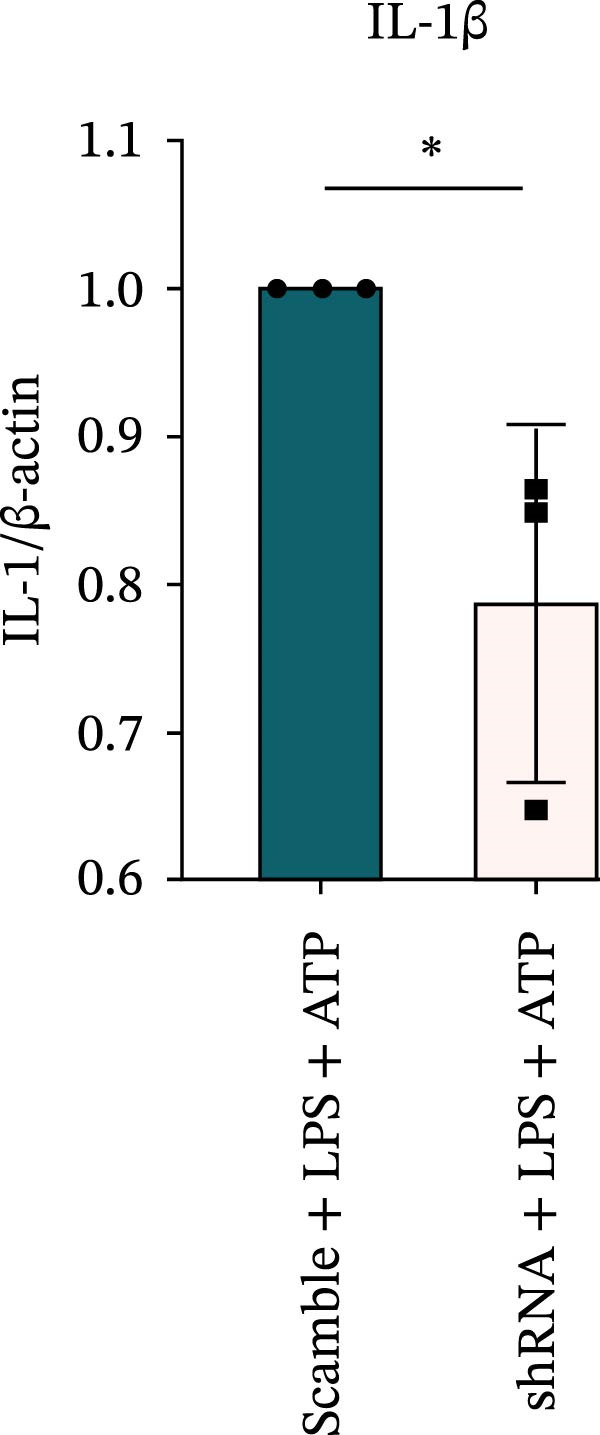
(G)

**Figure:**
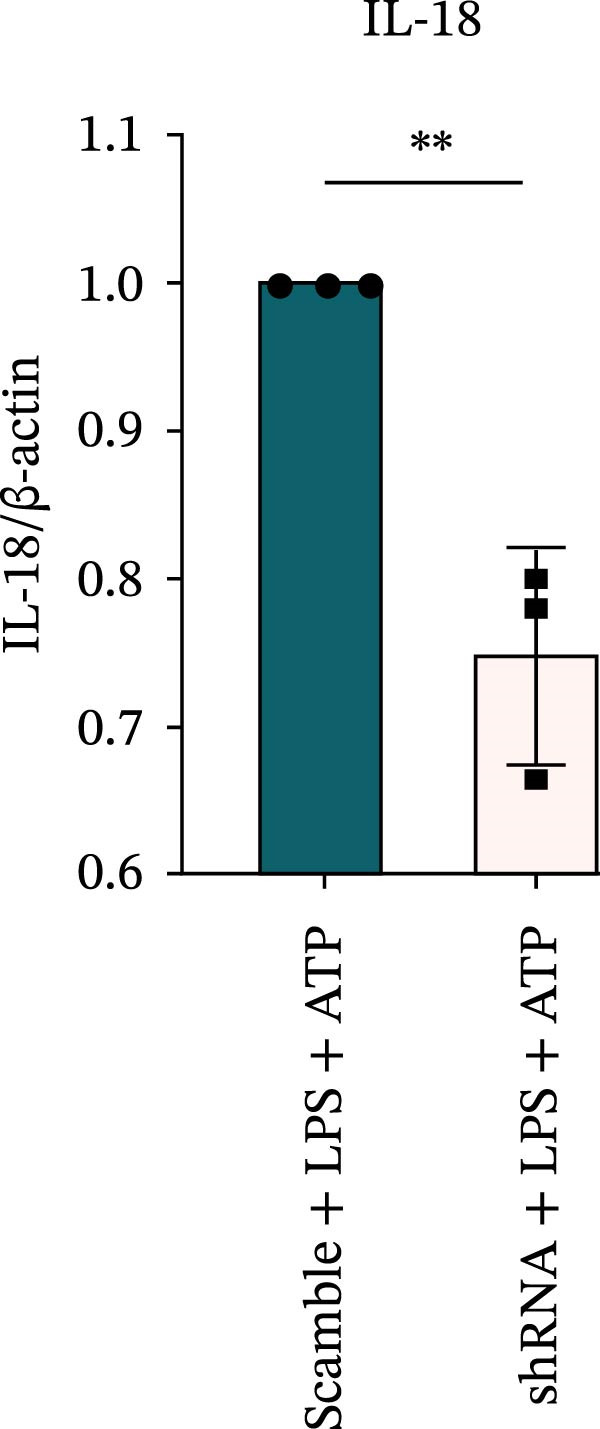
(H)

**Figure:**
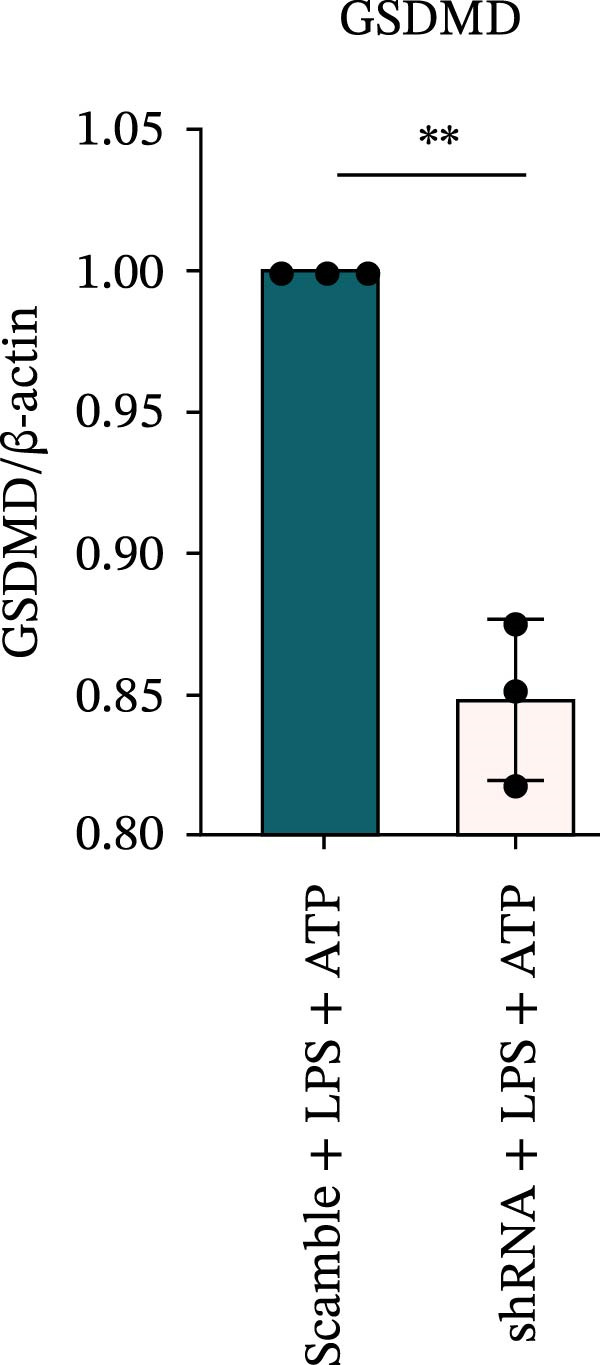
(I)

**Figure:**
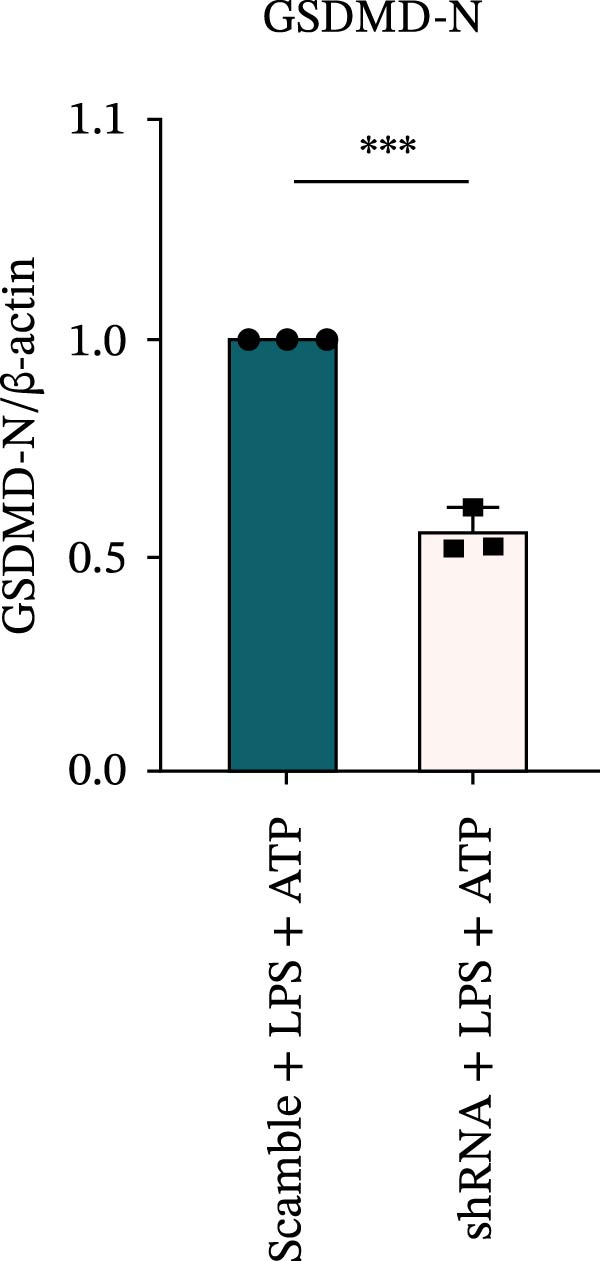
(J)

## 4. Discussion

The dental pulp is a highly vascularized tissue, and vascular ECs play a crucial role in the onset and progression of pulpitis [33]. However, owing to the lack of collateral circulation, the pulp has limited ability to resist infection and inflammation, which also increases the difficulty of pulpitis treatment [34]. During pulpitis, ECs may undergo pyroptosis, thereby contributing to the amplification of inflammation, although their specific role has not yet been fully elucidated [35]. Therefore, investigating the mechanisms underlying the activation of pyroptosis in pulp ECs is of great significance for revealing the pathological processes of pulpitis and guiding early intervention and targeted therapy. In this study, we found that ECs accounted for a relatively high proportion of pulp cells. Furthermore, the inflammatory microenvironment was shown to regulate EZH2 expression, which in turn activated the NLRP6 inflammasome, upregulated the release of downstream inflammatory factors, and ultimately promoted pyroptosis.

The vascular system of the dental pulp accounts for ~40% of the total tissue volume [34]. ECs, as the principal component of the vessel wall, play a critical role in maintaining tissue homeostasis and mediating pathological processes [11]. Based on publicly available scRNA‐seq data of pulp tissue, this study identified ECs and their subpopulation characteristics within the overall cellular atlas. We found that ECs constitute a relatively large proportion of pulp cells and exhibit diverse subcluster distributions, suggesting that the rich vascular network of the pulp may regulate the local inflammatory environment through different EC subsets. For example, previous studies have reported that in specific EC subtypes, such as capillary ECs, transendothelial migration and contact with immune cells may enhance the immunoregulatory functions of ECs, including antigen presentation or the secretion of inflammatory mediators, thereby influencing the local inflammatory microenvironment [36]. Together, these findings further investigated the role of ECs in pulpitis and their involvement in programmed cell death. Given that the pulp vasculature is predominantly microvascular in nature, the use of EOMA cells, which retain microvascular endothelial characteristics, provides a reasonable in vitro surrogate to investigate pulpitis‐associated endothelial inflammation [9, 32, 37].

In pulpitis, the vascular density and distribution of ECs in healthy pulp are markedly higher than those in irreversible pulpitis tissue, suggesting that EC injury or death may occur during disease progression [11, 35]. ECs exert critical immunomodulatory functions within the dental pulp, and dysregulation of EC death programs may directly reshape the local inflammatory microenvironment, consistent with the immune‐related enrichment observed in our transcriptomic analyses [36, 38, 39]. Increasing evidence indicates that pyroptosis plays a critical role in pulpitis by directly coupling regulated cell death with inflammatory amplification within the pulp tissue [35, 40]. By integrating publicly available microarray datasets, our study found that DEGs in pulpitis were significantly enriched in pyroptosis‐related pathways, indicating that pyroptosis may represent an important form of cell death during disease progression. Accordingly, elucidating the key molecular regulators of EC fate under inflammatory conditions is important for a better understanding of the occurrence and progression of pulpitis. Our previous work demonstrated that EZH2 participates in the regulation of EC fate under inflammatory conditions [32]. In this context, our transcriptomic analyses showed that PRGs were highly expressed in ECs and exhibited coordinated changes following EZH2 deficiency, with enrichment across arterial, venous, and capillary EC subpopulations. These findings suggest that EC subsets may display differential sensitivity to pyroptosis in the inflammatory pulp environment. In addition, our in vitro experiments demonstrated that LPS‐induced inflammatory stimulation markedly upregulated the expression of GSDMD‐N, ASC, Cle‐caspase‐1, and the downstream cytokines IL‐1β and IL‐18. Notably, pyroptosis may interact with other forms of programmed cell death, such as ferroptosis, further increasing the mechanistic complexity of pulpitis pathology [33]. Unlike apoptosis or ferroptosis, pyroptosis is characterized by the release of inflammatory mediators, a feature that may confer a unique amplifying effect in pulpitis [41]. Collectively, these findings suggest that pyroptosis is closely associated with the occurrence and progression of pulpitis and may represent a potential therapeutic target.

The results of this study showed that there was a significant association between DEGs in pulpitis and histone methylation‐related genes, suggesting that epigenetic modifications might play a role in pyroptosis of ECs in pulpitis [42, 43]. It has been reported that histone methylation can regulate the activation status of inflammation‐related genes, thereby influencing the inflammatory process [44, 45]. As a key histone methyltransferase, the regulatory role of EZH2 in pulpitis has attracted attention [46, 47]. Consistent with this, previous studies have reported that EZH2 inhibition can delay the development of pulpitis while reducing the expression of the key pyroptosis molecule GSDMD and regulating the secretion of inflammatory factors[48–50]. In this study, we further focused on ECs of pulpitis and found that endothelium‐specific EZH2 deletion could significantly alter the expression pattern of PRGs. In vitro experiments also indicated that the EZH2 inhibitor GSK126 could down‐regulate the expression of pyroptosis molecules in ECs, suggesting that EZH2 might affect the progression of pulpitis by regulating pyroptosis in ECs. These results provide a molecular basis for further investigation of EC‐specific inflammatory mechanisms in pulpitis.

Our analyses revealed that PRGs highly correlated with EZH2 were primarily enriched in the NOD‐like receptor signaling pathway, with NLR family members serving as key components. Although the role of NLRP3 in pulpitis has been extensively investigated, the functions of other NLR family members remain unclear [51, 52]. We found that under inflammatory conditions, NLRP6 expression in ECs was markedly upregulated and showed strong colocalization with ASC and Caspase‐1, indicating inflammasome formation and activation. Notably, NLRP6 expression decreased upon EZH2 inhibition, suggesting that EZH2 may contribute to inflammation‐associated endothelial pyroptosis by regulating NLRP6. This finding complements previous research focused primarily on NLRP3 and highlights the potential role of NLRP6 in the pathogenesis of pulpitis. Previous studies have shown that NLRP6 can assemble inflammasomes and drive pyroptosis in inflamed tissues, and its promoter region is enriched for EZH2 and H3K27me3, consistent with the correlation between EZH2 and NLRP6 expression observed in our study [19, 53]. Further experiments demonstrated that NLRP6 knockout under inflammatory conditions significantly reduced the expression of pyroptosis‐related molecules, directly supporting its critical role in endothelial pyroptosis and inflammatory responses. Collectively, these findings not only expand our understanding of the regulatory mechanisms of endothelial pyroptosis in pulpitis but also underscore the function of the NLRP6 inflammasome in amplifying local inflammation, providing a theoretical basis for exploring the EZH2/NLRP6 axis as a potential therapeutic target.

In summary, the present study suggests that EZH2‐mediated activation of the NLRP6 inflammasome may represent a key molecular mechanism driving EC pyroptosis in pulpitis. Inhibition of EZH2 or NLRP6 activity holds the potential to mitigate inflammation‐induced cellular damage, preserve endothelial function, and maintain pulp microenvironment homeostasis, thereby creating favorable conditions for pulp protection and regeneration. Based on this mechanism, the future development of small‐molecule inhibitors targeting EZH2 or NLRP6, alone or in combination with anti‐inflammatory or antioxidant therapies, may provide novel strategies and a theoretical basis for multitargeted treatment of pulpitis.

Despite a systematic analysis of EC pyroptosis and the potential EZH2/NLRP6 axis in pulpitis, several limitations should be noted. Mechanistic studies were performed in EOMA cells due to the limited availability of primary dental pulp microvascular ECs. EZH2^fl/fl^;Tie2‐Cre^+/−^ mice were used for transcriptomic analyses, while experimental pulpitis models were not established. In addition, EZH2 binding to the NLRP6 promoter and H3K27me3 enrichment were not directly assessed, and clinical pulp samples were not included. Future studies will be required to address these issues.

## 5. Conclusion

This study revealed that ECs undergo pyroptosis during pulpitis and that LPS stimulation induces this process in EOMA cells. Importantly, EZH2 promotes EC pyroptosis by activating the NLRP6 inflammasome, while inhibition of EZH2 or deletion of NLRP6 markedly attenuates this effect. These findings underscore the functional relevance of the EZH2/NLRP6 axis and provide new insights into the molecular mechanisms of pulpitis‐associated cell death.

## Author Contributions

Conceptualization: Yi Huang. Methodology, formal analysis, investigation, data curation, writing – original draft preparation, visualization: Weilin Zhou and Weili Huang. Software: Weili Huang and Hongjing You. Validation: Hongjing You. Resources: Zhixi Huang and Jingye Zhou. Writing – review and editing: Yi Huang. Supervision: Yi Huang. Project administration: Zhixi Huang and Jingye Zhou. Funding acquisition: Yi Huang.

## Funding

This research was funded by the Guangzhou Science and Technology Planning Project (Grant 2023A03J0590).

## Disclosure

All authors have read and agreed to the published version of the manuscript.

## Ethics Statement

The animal study protocol was approved by the Ethics Committee of the School of Pharmaceutical, Guangzhou University of Chinese Medicine (SYXK(YUE) 2019‐0202).

## Conflicts of Interest

The authors declare no conflicts of interest.

## Supporting Information

Additional supporting information can be found online in the Supporting Information section.

## Supporting information


**Supporting Information** Table S1. Main cell types of dental pulp and their marker genes. Table S2. Main endothelial cell subtypes of dental pulp and their marker genes. Table S3. List of 19 histone methylation‐related genes included in the analysis. Table S4. List of 33 pyroptosis‐related genes included in the analysis. Figure S1. Polymerase chain reaction verification of mouse gene expression.

## Data Availability

The data that support the findings of this study are available from the corresponding author upon reasonable request.
