# Supplementary material for: EZH2‐Mediated Epigenetic Modifications Induce Pyroptosis in Dental Pulp Endothelial Cells via Activation of NLRP6 Inflammasome During Pulpitis Development
Source: Mediators Inflamm. 2026 Apr 8;2026:5876995. doi: 10.1155/mi/5876995 (PMC13058821; doi:10.1155/mi/5876995)
Supplement: Supplementary file 1 — Supporting Information Table S1. Main cell types of dental pulp and their marker genes. Table S2. Main endothelial cell subtypes of dental pulp and their marker genes. Table S3. List of 19 histone methylation‐related genes included in the analysis. Table S4. List of 33 pyroptosis‐related genes included in the analysis. Figure S1. Polymerase chain reaction verification of mouse gene expression. [file MI-2026-5876995-s001.pdf]

Table S1: Main cell types of dental pulp and their marker genes

Cell types	Marker
T cell natural killer cell (T-NK-Cell)	CD3D
B-Cell	CD79A
Epithelial	KRT14
Endothelial	VWF
Mesenchymal Stem Cell (MSC)	FRZB
Fibroblast	LUM
Odentoblast	DMP1
Glial-Cell	PLP1
Myeloid	C1QC

Table S2: Main cell types of dental pulp endothelial cells and their marker genes

Endothelial Cell types	Marker
Arteries	GJA5, FN1
Vein	ACKR1, SELE
Capillary	CA4
Lymphatic	FABP4, FABP5, MMRN1
CD34+	CD34+

Tables S3: The list of 19 histone methylation–related genes

HGNC approved symbol	Function
ASH1L	Histone modification write
CARM1	Histone modification write
DOT1L	Histone modification write
EHMT2	Histone modification write
JARID2	Histone modification write cofactor
JMJD1C	Histone modification erase
JMJD6	Histone modification erase
KDM1B	Histone modification erase
KDM2A	Histone modification erase
KDM3B	Histone modification erase
KDM4A	Histone modification erase
KDM5B	Histone modification erase
KDM6B	Histone modification erase
NSD1	Histone modification write
PRMT1	Histone modification write
PRMT5	Histone modification write
SETD1A	Histone modification write
SETD2	Histone modification write
SUV39H2	Histone modification write

Table S4: The list of 33 pyroptosis-related genes

Gene	Type
AIM2	writers
CASP1	writers
CASP3	writers
CASP4	writers
CASP5	erasers
CASP6	erasers
CASP8	readers
CASP9	readers
ELANE	readers
GPX4	readers
GSDMA	readers
GSDMB	readers
GSDMC	readers
GSDMD	readers
GSDME	readers
IL18	readers
IL1B	readers
IL6	readers
NLRC4	readers
NLRP1	readers
NLRP2	readers
NLRP3	readers
NLRP6	readers
NLRP7	readers
NOD1	readers
NOD2	readers
PJVK	readers
PLCG1	readers
PRKACA	readers
PYCARD	readers
SCAF11	readers
TIRAP	readers
TNF	readers

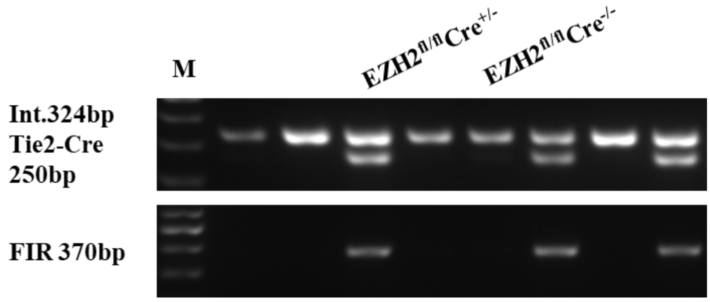

Figure S1: Polymerase chain reaction verification of mouse gene expression.
